# Centipede Protein-Laden Natural Nanocapsule Hybrid Hydrogel Mediates Sustained Bioactive Release for Synergistic Regeneration of Diabetic Foot Ulcers

**DOI:** 10.3390/ph19081289

**Published:** 2026-08-14

**Authors:** Shun Lv, Jian Hu, Huan Chen, Minyu Zhu, Wei Jin, Qiyin Liu, Qianqian Zhang, Yinghua Zhang, Ying Li, Zhengqi Dong

**Affiliations:** 1 State Key Laboratory for Quality Ensurance and Sustainable Use of Dao-Di Herbs, Institute of Medicinal Plant Development, Chinese Academy of Medical Sciences, Peking Union Medical College, Beijing 100193, China; 13304371699@163.com (S.L.); 15170362242@163.com (J.H.); 15030413919@163.com (H.C.); 18848256164@163.com (M.Z.); jinw0205@163.com (W.J.); 19923421882@163.com (Q.L.); zqqhdsajvnks@163.com (Q.Z.); 2 School of Pharmacy, Qinghai University, Xining 810016, China; 3 Jilin Provincial Academy of Chinese Medicine, Changchun 130012, China; zhangyinghua0214@126.com

**Keywords:** diabetic foot ulcers, hydrogel, nanocapsule, centipede-derived components, wound healing

## Abstract

**Background**: Diabetic foot ulcers (DFUs) are chronic wounds characterized by persistent inflammation, oxidative stress, impaired angiogenesis, and defective extracellular matrix remodeling. Current therapeutic approaches remain insufficient for refractory diabetic wounds due to limited drug retention and inadequate regulation of the wound microenvironment. This study aimed to develop a centipede-derived protein fraction-loaded nanocapsule hybrid hydrogel for sustained bioactive delivery and diabetic wound repair. **Methods**: A bioactive protein fraction was isolated from processed medicinal centipede material and screened using cellular compatibility assays. The selected tropomyosin-containing protein fraction was incorporated into nanocapsules and subsequently integrated into a gallic acid-modified polyacrylamide hydrogel matrix. The physicochemical properties, antioxidant activity, rheological behavior, and protein release characteristics of the nanocapsule-hydrogel system were systematically evaluated. A streptozotocin-induced diabetic rat wound model was established to assess therapeutic efficacy through wound closure analysis, histological staining, and immunohistochemical evaluation. **Results**: The prepared nanocapsules exhibited a spherical morphology, nanoscale size distribution, and sustained protein delivery capability. The PAM-GA hydrogel demonstrated antioxidant activity, injectability, shear-thinning behavior, self-healing ability, and enhanced retention of protein release. In vivo experiments showed that the PT@NC@PAM-GA hydrogel significantly accelerated wound closure and promoted tissue regeneration, accompanied by enhanced angiogenesis, collagen deposition, and reduced inflammatory responses. **Conclusions**: The centipede-derived protein fraction-loaded nanocapsule hybrid hydrogel effectively integrates bioactive protein delivery with a multifunctional hydrogel matrix, providing a potential strategy for sustained treatment of diabetic foot ulcers.

## 1. Introduction

Among diabetic complications, DFUs (diabetic foot ulcers) pose formidable challenges, precipitating lower limb ulcerations resistant to healing [1,2]. Microvascular anomalies, secondary to prolonged hyperglycemia, culminate in intractable ulcers, with approximately 20% of DFU cases culminating in amputations, profoundly impacting patient well-being [3,4].

Contemporary therapeutic modalities encompass glycemic regulation [5], foot pressure alleviation [6], debridement and dressings [7], antibiotic regimens [8], local ulcer interventions [9], and surgical procedures [10].

Although glycemic control is paramount for all diabetes management strategies, it remains particularly pivotal for DFUs management [11,12]. However, dietary restrictions and medication adherence pose formidable obstacles [13]. Foot pressure mitigation aims to facilitate ulcer healing by minimizing ulcerative regions’ pressure burden, yet compromised mobility in infected or ulcerated foot regions poses challenges [14]. Debridement and dressings constitute the standard of care for ulcer treatment, but persistent hyperglycemia impedes wound healing, augmenting the risk of re-infection and necessitating iterative surgeries. Antibiotic regimens combat infections, but prolonged use fosters microbial resistance and necessitates multidrug interventions, exacerbating the risk of drug-resistant infections and adverse effects [15,16]. Innovative local ulcer interventions such as growth factor application, stem cell therapy, and negative pressure wound therapy (NPWT) show promising results, but their prohibitive costs render them inaccessible to many [17,18]. Surgical interventions, including debridement, amputation, and peripheral vascular reconstruction, may be warranted in severe cases but pose heightened risks in diabetic patients [19].

In recent years, natural medicinal products have gained increasing attention for their potential in tissue regeneration and chronic wound repair [20]. Centipede is a traditional Chinese medicinal material containing various bioactive components, including proteins, peptides, enzymes, and amino acids. Previous studies have suggested that centipede-derived components may possess anti-inflammatory, microcirculation-associated, and tissue-repair-related biological activities [21,22]. However, the specific bioactive components responsible for these effects remain insufficiently characterized.

In this study, centipede-derived protein fractions were isolated and screened based on their wound-healing-related cellular activity. The selected protein fraction was used as the bioactive component for subsequent nanocapsule and hydrogel construction. As a proteinaceous therapeutic component, it may exhibit limitations including instability, rapid diffusion, and insufficient local retention in the complex diabetic wound microenvironment. Therefore, an appropriate delivery strategy is required to improve its stability and therapeutic performance.

Although current therapeutic strategies have improved wound management to some extent, their efficacy remains limited in chronic diabetic wounds due to persistent inflammation, excessive oxidative stress, impaired angiogenesis, poor extracellular matrix remodeling, and insufficient local retention of therapeutic agents. Therefore, there is still a need to develop multifunctional wound dressings capable of providing sustained bioactive release and regulating the diabetic wound microenvironment.

To overcome the instability and rapid release of centipede-derived bioactive proteins, nanotechnology has been increasingly applied to improve the stability, local retention, and sustained release of natural bioactive components [23]. Hydrogels are also widely used in wound healing, drug delivery, and tissue engineering because of their good biocompatibility, tunable physicochemical properties, moist wound-healing environment, and ability to conform to irregular wound surfaces [24].

In this study, we developed a centipede protein-loaded nanocapsule hybrid hydrogel for diabetic wound repair. First, bioactive protein fractions were isolated from centipede-derived material and screened for wound-healing-related cellular activity. The selected protein component was then encapsulated into nanocapsules to improve its stability and sustained release behavior, forming protein-loaded nanocapsules (PT@NC). Subsequently, the nanocapsules were incorporated into a gallic acid-modified polyacrylamide hydrogel matrix to obtain the nanocapsule hybrid hydrogel PT@NC@PAM-GA.

**Figure 1:**
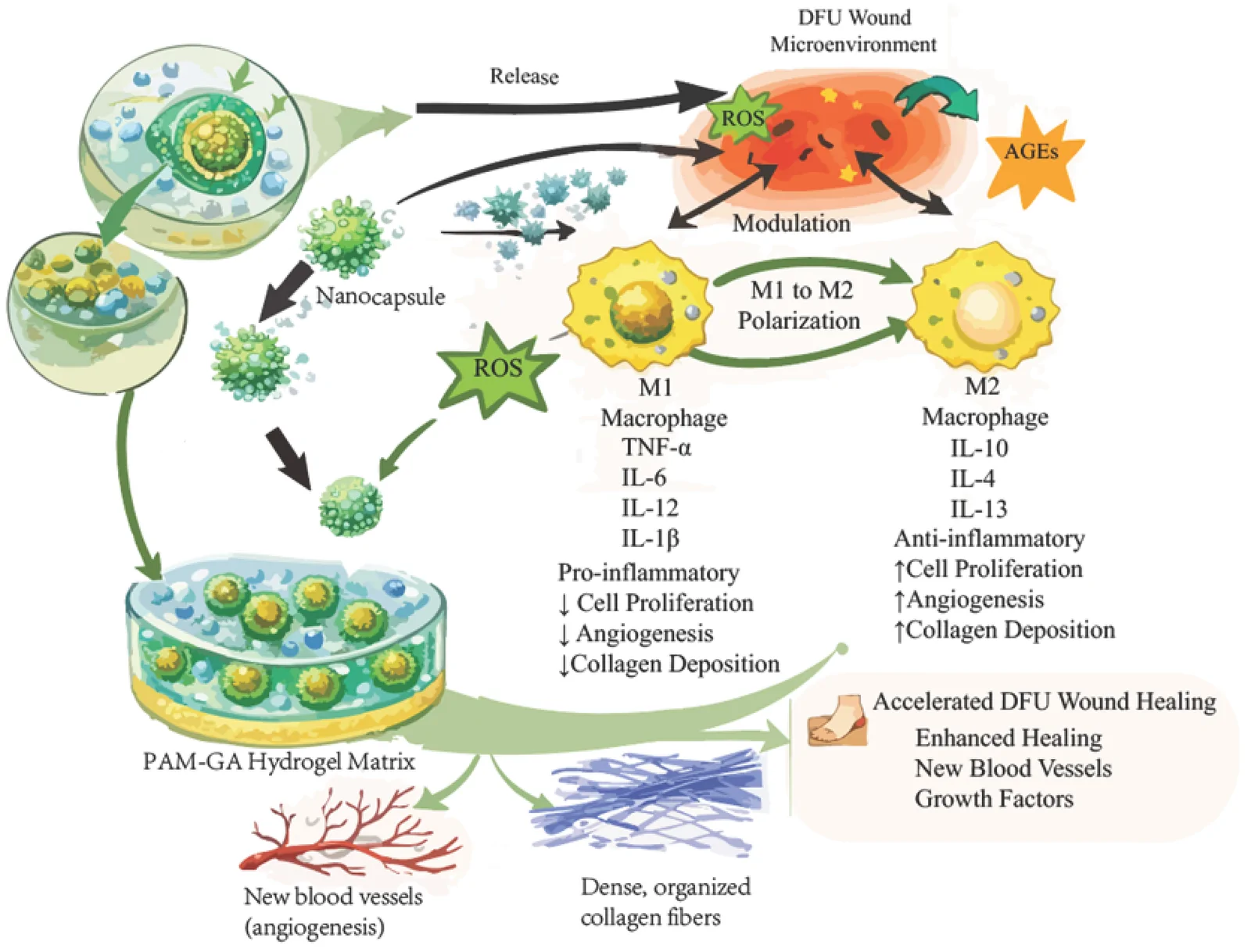
Schematic illustration of the mechanism by which the PT@NC@PAM-GA nanocomposite hydrogel promotes diabetic foot ulcer (DFU) healing. Black arrows indicate nanocapsule formation/release and interactions within the DFU wound microenvironment, whereas green arrows indicate beneficial modulatory and regenerative effects. ↑ and ↓ indicate increases and decreases, respectively.

The novelty of this work lies in the combination of a screened centipede-derived bioactive protein fraction with a multifunctional nanocapsule-hydrogel delivery platform. This system integrates sustained protein release from nanocapsules with the antioxidant, injectable, self-healing, and wound-covering properties of the PAM-GA hydrogel matrix. As illustrated in Figure 1, PT@NC@PAM-GA was designed to regulate the diabetic wound microenvironment by reducing oxidative and inflammatory stress, supporting angiogenesis, and promoting collagen deposition. The therapeutic efficacy of this integrated system was further evaluated in a diabetic wound rat model, providing a potential strategy for sustained local treatment of diabetic foot ulcers.

## 2. Results

### 2.1. Proteins Composition Identification

As shown in Table 1, the purified protein fractions were analyzed by mass spectrometry and matched against available UniProt protein entries. Due to the limited reference proteome annotation for Scolopendra subspinipes mutilans, some peptide sequences were assigned to homologous proteins from closely related centipede species available in the database, such as *Scolopendra viridis* and *Hemiscolopendra marginata*. Therefore, the species names shown in Table 1 represent the closest available database matches rather than different biological source materials.

Based on mass spectrometry peptide matching and UniProt database comparison, six major protein-related fractions were assigned, including Calglandulin, Tropomyosin, Myosin light chain 2, 62% Actin + 25% Nucleoredoxin-like protein 2, 75% Actin + 5% Glyceraldehyde-3-phosphate dehydrogenase, and 58% Actin + 7% Glyceraldehyde-3-phosphate dehydrogenase. These assignments represent the predominant peptide matches detected in each fraction rather than definitive identification of single purified proteins. The percentage values included in the fraction names represent the relative peptide assignment proportions derived from mass spectrometry analysis rather than the absolute protein composition or abundance ratio of individual proteins.

Among these identified protein-related fractions, the tropomyosin-containing fraction was further selected for subsequent nanocapsule construction based on its relatively favorable cellular compatibility and metabolic activity in preliminary screening experiments. It should be noted that the identified proteins were not considered centipede-specific proteins, but rather represent centipede-derived protein fractions identified through database-supported peptide matching and screened for their potential therapeutic application.

### 2.2. Biocompatibility Determination

NIH3T3 fibroblasts and HaCaT keratinocytes were selected as complementary cell models because fibroblasts and keratinocytes play distinct but essential roles in diabetic wound repair, including extracellular matrix remodeling and re-epithelialization, respectively. Cells without protein treatment were used as the cellular control group, while cells treated with different centipede-derived protein fractions were used as the experimental groups. The relative cell viability/metabolic activity was evaluated using the CCK-8 assay.

**Figure 2:**
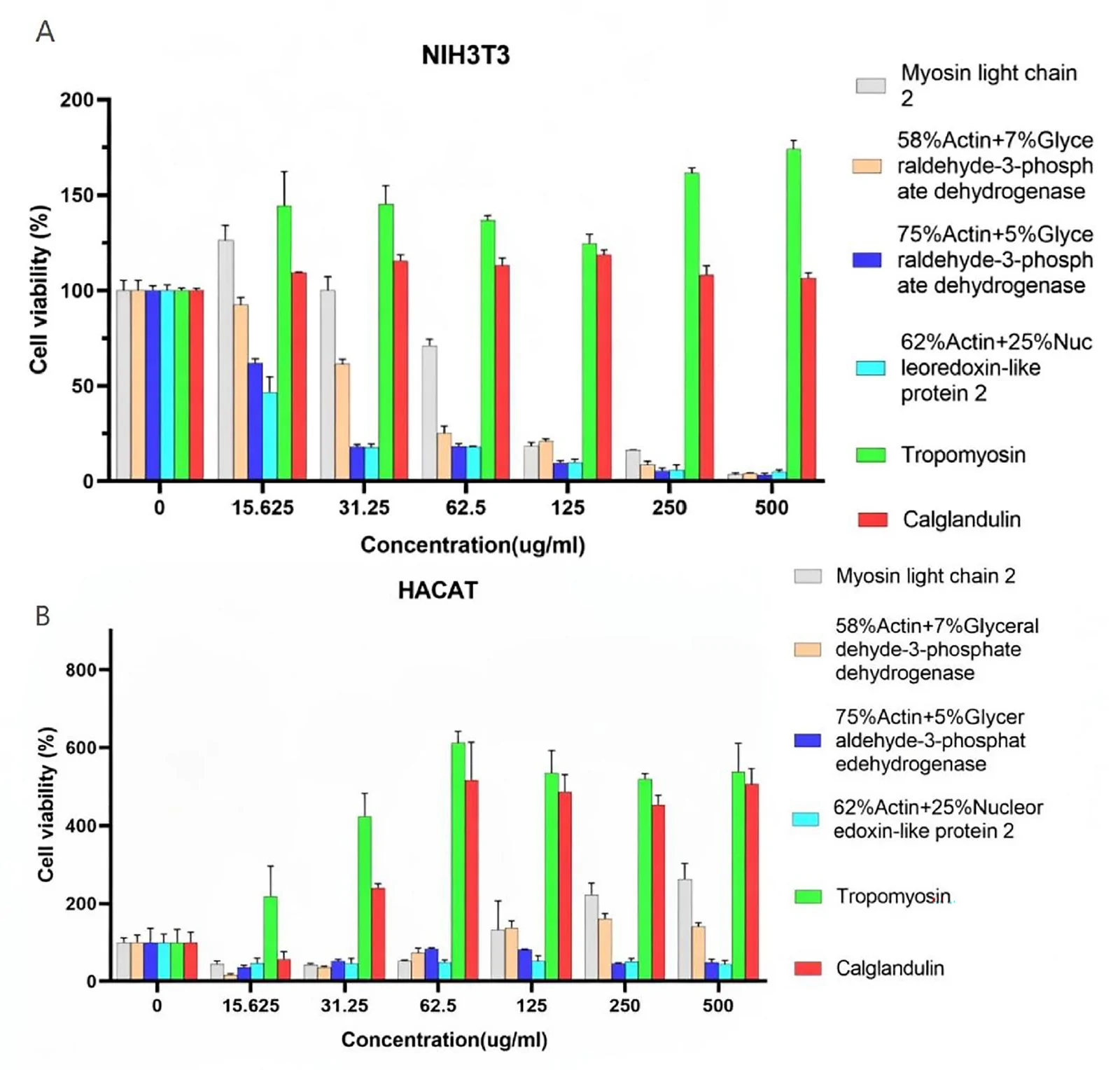
The cell bioactivity of purified proteins. (**A**). The cell bioactivity of purified proteins on NIH3T3 cells. (**B**). The cell bioactivity of purified proteins on HACAT cells. (Mean ± SD, *n* = 3).

As shown in Figure 2A,B, the six purified centipede-derived protein fractions showed concentration-dependent effects on the CCK-8-based viability of NIH3T3 and HaCaT cells. In NIH3T3 cells, tropomyosin and calglandulin maintained relatively high cell viability over the tested concentration range, whereas myosin light chain 2 and the actin-containing composite fractions showed decreased cell viability at higher concentrations. In HaCaT cells, tropomyosin and calglandulin induced a pronounced increase in CCK-8 signal, with relative values reaching approximately 500–600% of the control group at certain concentrations.

Because such high CCK-8 values are unusual in standard cell viability assays, these results should be interpreted cautiously. The CCK-8 assay primarily reflects cellular metabolic activity based on dehydrogenase-mediated reduction in tetrazolium salts and does not directly represent cell number. Therefore, the increased absorbance may reflect enhanced cellular metabolic activity, increased cell proliferation, or potential interactions between protein components and the detection system. Since cell-free interference controls containing protein samples and CCK-8 reagent without cells were not included in the original experimental design, direct effects of protein samples on the CCK-8 reaction cannot be completely excluded.

Accordingly, these results are described as enhanced CCK-8-based cell viability/metabolic activity rather than direct evidence of several-fold increases in cell proliferation. Among the tested protein fractions, the tropomyosin-containing fraction was selected for subsequent nanocapsule construction because it showed a relatively favorable compatibility profile in both NIH3T3 and HaCaT cells and provided a suitable candidate for further delivery-system development. The contribution of individual protein components requires further validation using purified proteins, recombinant proteins, and additional mechanistic assays.

Overall, the CCK-8 results provided preliminary information regarding the cellular compatibility of centipede-derived protein fractions. The selection of the tropomyosin-containing fraction was therefore based on its overall screening performance together with subsequent nanocapsule preparation, release characteristics, and in vivo wound-healing evaluation, rather than solely on the increased CCK-8 signal observed in HaCaT cells. Further validation using cell-free interference controls, direct cell counting, EdU incorporation assays, or live/dead staining will be valuable to clarify the biological effects of these protein fractions.

### 2.3. Structural and Morphological Characterization of Nanocapsules

Different feeding amounts of N-acryloyl succinimide (NAS) were tested to optimize the acrylation degree of tropomyosin before nanocapsule preparation. Among the tested formulations, the one-fold and two-fold NAS feeding groups showed effective acrylation and were selected for subsequent nanocapsule synthesis. When three-fold or higher NAS feeding amounts were used, the fluorescence intensity showed little additional change after gradient dilution, suggesting that the modification level reached a plateau at the two-fold NAS feeding amount. The grafting rates of the one-fold and two-fold NAS feeding formulations were 39.74 ± 4.61% and 71.50 ± 2.69%, respectively. The resulting tropomyosin-loaded nanocapsules were named nTro and 2nTro, respectively.

**Figure 3:**
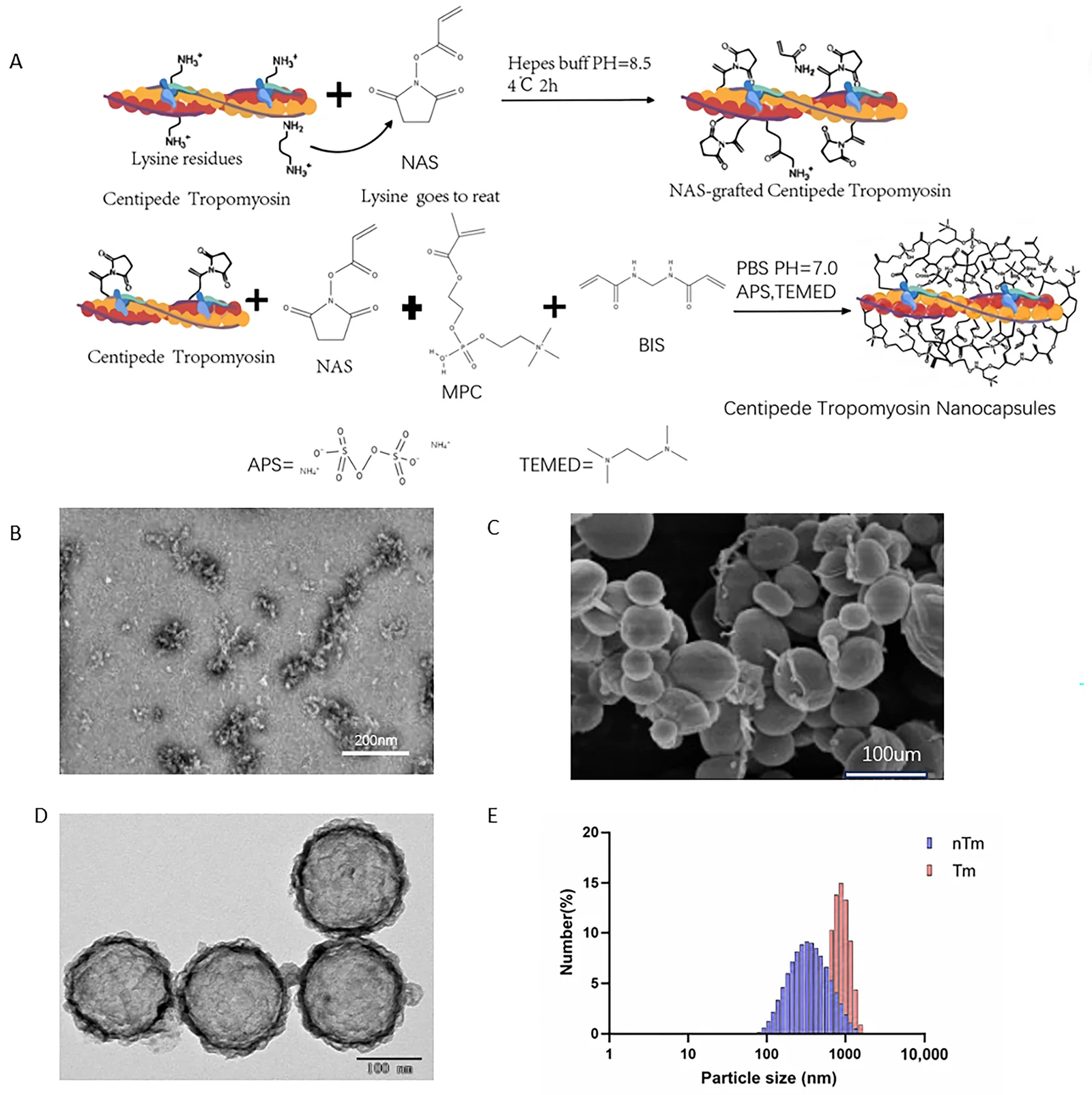
Morphological and particle size characterization of protein-loaded nanocapsules. (**A**) Schematic illustration of the preparation process of tropomyosin-loaded nanocapsules. (**B**,**C**) SEM images showing the surface morphology, particle distribution, and dispersion of the prepared nanocapsules at different magnifications. (**D**) TEM image showing the spherical morphology, internal structure, and nanoscale characteristics of the nanocapsules. (**E**) Particle size distribution analysis of blank nanocapsules (nTm) and tropomyosin-loaded nanocapsules (Tm).

The preparation process of the tropomyosin-loaded nanocapsules is illustrated in Figure 3A. Following NAS-mediated acrylation of tropomyosin, the modified protein was incorporated into a polymeric nanocapsule system through free-radical polymerization initiated by APS and TEMED in the presence of MPC and BIS.

The morphology of the prepared nanocapsules was characterized by SEM and TEM analysis. As shown in Figure 3B,C, the SEM images revealed that the nanocapsules exhibited relatively smooth surfaces, uniform particle distribution, and no obvious aggregation. TEM analysis further demonstrated spherical nanocapsules with a clear internal structure and good dispersion (Figure 3D). These results indicated the successful formation of a stable nanoscale carrier structure. The loading efficiency and encapsulation efficiency of the nanocapsules were 37.1% and 74.1%, respectively.

The particle size distribution analysis further confirmed the nanoscale characteristics of the prepared nanocapsules (Figure 3E). Compared with blank nanocapsules (nTm), tropomyosin-loaded nanocapsules (Tm) exhibited a shifted particle size distribution, indicating successful incorporation of tropomyosin into the nanocapsule structure. The increased particle size after protein loading further supported the formation of a protein-containing nanocapsule system.

The chemical characteristics of the nanocapsule system were further investigated by UV–visible spectroscopy, Fourier-transform infrared spectroscopy (FTIR), and ^1^H nuclear magnetic resonance (^1^H NMR) spectroscopy. The corresponding characterization results are provided in Supporting Figure S1. These analyses showed characteristic signals associated with the major components involved in nanocapsule formation, supporting the successful incorporation of the protein component and polymerization-related materials into the nanocapsule system.

### 2.4. In Vitro Protein Release Study of Nanocapsules

**Figure 4:**
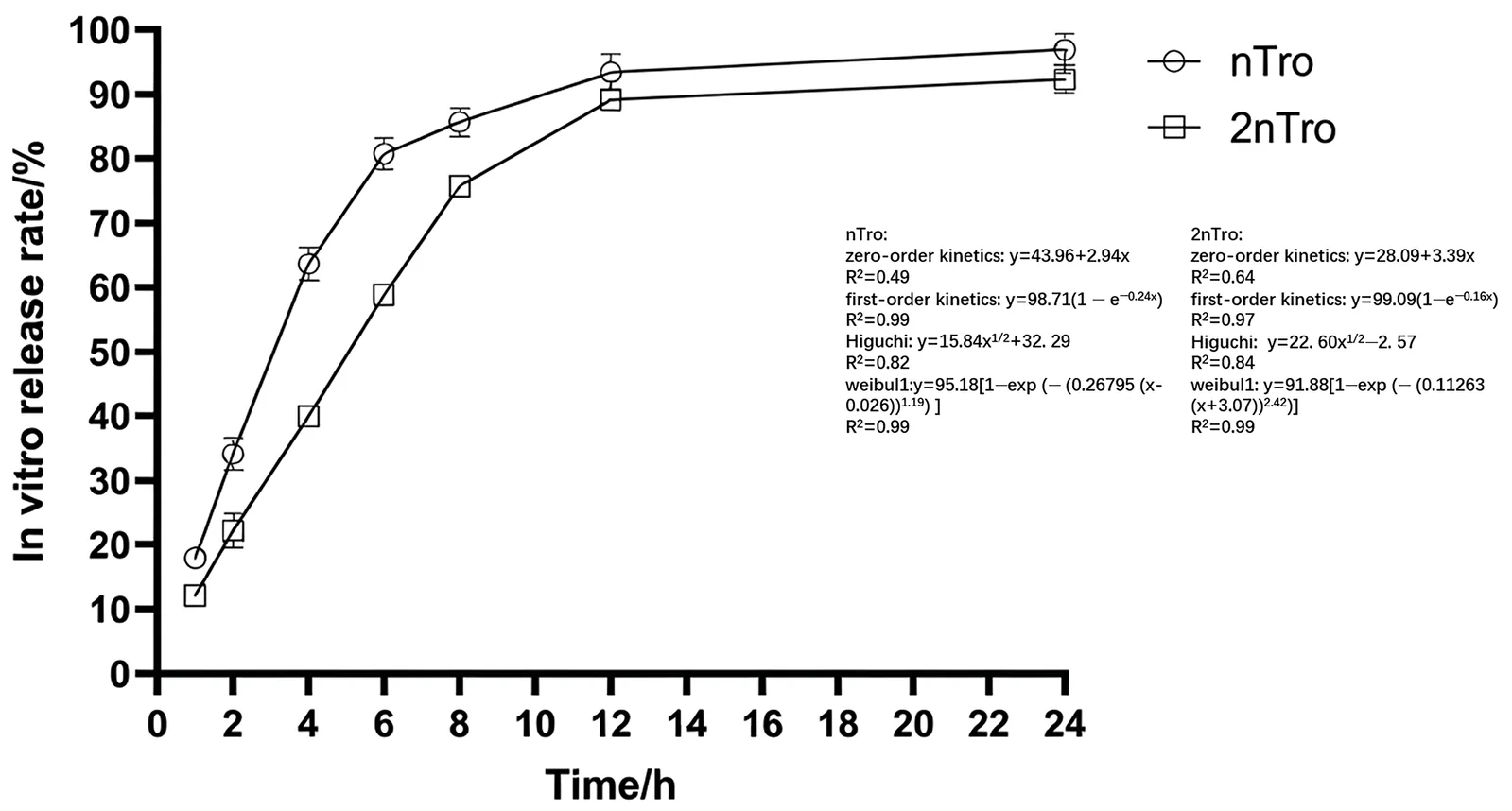
In vitro release profiles of centipede-derived tropomyosin protein from two nanocapsule formulations (nTro and 2nTro).

The in vitro release behavior of centipede-derived tropomyosin protein from two nanocapsule formulations, nTro and 2nTro, was evaluated over 24 h (Figure 4). Both formulations exhibited a sustained release profile, with the cumulative release percentage increasing gradually over time and approaching a plateau at 24 h.

The release rate was formulation-dependent. The nTro group showed a faster release profile, reaching approximately 95% cumulative protein release at 12 h and approximately 98% at 24 h. In contrast, the 2nTro formulation exhibited a relatively slower release profile, reaching approximately 90% release at 12 h and approximately 93% at 24 h. The release rate followed the order nTro > 2nTro at most time points, indicating that the increased formulation density in 2nTro may retard protein diffusion from the nanocapsules.

To further analyze the release behavior, four kinetic models, including zero-order, first-order, Higuchi, and Weibull models, were used to fit the release data. The coefficient of determination (R^2^) was used to evaluate the goodness of fit.

For the nTro group, the zero-order model showed a poor fit, with the fitting equation y = 43.96 + 2.94x and R^2^ = 0.49. The first-order model showed a high goodness of fit, with the equation y = 98.71(1 − e^−0.24x^) and R^2^ = 0.99. The Higuchi model showed a moderate fit, with the equation y = 15.84x^1/2^ + 32.29 and R^2^ = 0.82, indicating that simple diffusion alone could not fully explain the release process. The Weibull model also showed a high goodness of fit, with the equation y = 95.18[1 − exp{−(0.26795(x – 0.026))^1.19^}] and R^2^ = 0.99. These results suggest that the release of tropomyosin protein from nTro may involve concentration-dependent release behavior together with a complex diffusion process.

For the 2nTro group, the zero-order model showed a moderate fit, with the fitting equation y = 28.09 + 3.39x and R^2^ = 0.84. The first-order model fitted the release data well, with the equation y = 99.09(1 − e^−0.26x^) and R^2^ = 0.97. The Higuchi model also showed a moderate fit, with the equation y = 22.60x^1/2^ − 2.57 and R^2^ = 0.84. Among the tested models, the Weibull model provided the highest goodness of fit for 2nTro, with the equation y = 91.88[1 − exp{−(0.11263(x + 3.07))^1.25^}] and R^2^ = 0.99. This result indicates that the release behavior of 2nTro was better described by a Weibull-type model, suggesting a multi-phase release process that may involve initial diffusion followed by slower protein release from the nanocapsule network.

Overall, these results confirmed that the nanocapsules enabled sustained release of centipede-derived tropomyosin protein within 24 h, and that the 2nTro formulation showed a slower release rate than nTro.

### 2.5. Structural Characterization, Antioxidant Activity and Rheological Properties of Hydrogels

**Figure 5:**
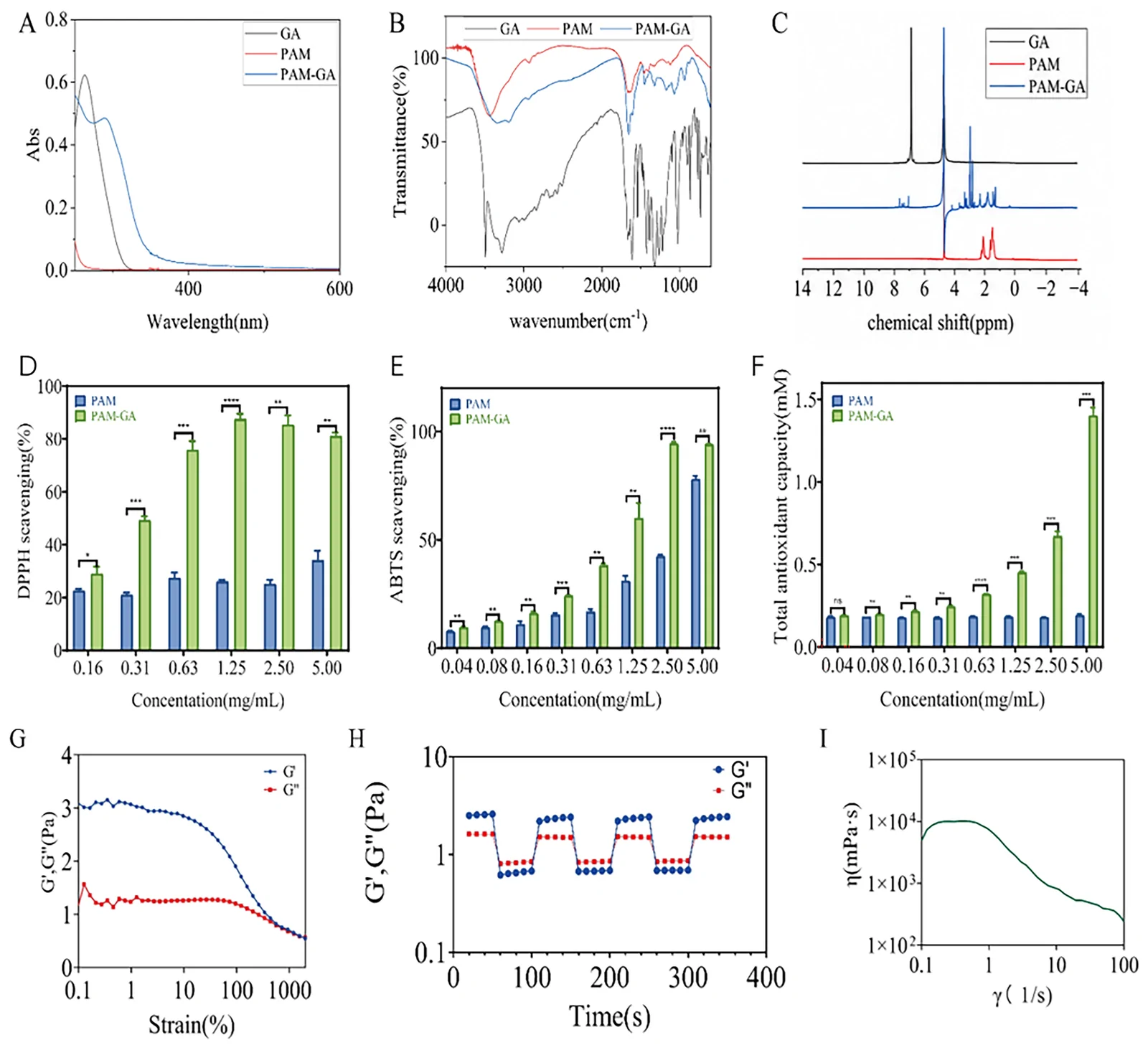
Physicochemical characterization, antioxidant activity, and rheological properties of the PAM-GA hydrogel system. (**A**) UV–visible absorption spectra of GA, PAM, and PAM-GA. (**B**) FTIR spectra showing characteristic functional groups and interactions among GA, PAM, and PAM-GA. (**C**) ^1^H NMR spectra of GA, PAM, and PAM-GA recorded in D_2_O, showing characteristic proton signals associated with the polymer backbone and GA moieties. (**D**) DPPH radical scavenging activity of PAM and PAM-GA at different concentrations. (**E**) ABTS radical scavenging activity of PAM and PAM-GA at different concentrations. (**F**) Total antioxidant capacity expressed as Trolox equivalent. (**G**) Strain sweep rheological analysis showing storage modulus and loss modulus. (**H**) Step-strain recovery test demonstrating self-healing behavior. (**I**) Apparent viscosity as a function of shear rate demonstrating shear-thinning behavior. Data in (**D**–**F**) are presented as mean ± SD (*n* = 3). Statistical significance compared with the PAM group at the same concentration: ns, not significant; * *p* < 0.05; ** *p* < 0.01; *** *p* < 0.001; **** *p* < 0.0001.

A. UV-Vis absorption spectra of GA, PAM, and PAM-GA(polyacrylamide-gallic acid hydrogel). B. FTIR spectra showing the functional group interactions in GA, PAM, and PAM-GA. The ^1^H NMR spectrum of PAM-GA showed characteristic signals from both PAM and GA (Figure 5C). The broad peaks at approximately 1.5–2.4 ppm were attributed to the aliphatic backbone protons of PAM, while the aromatic proton signals at approximately 7.05–7.25 ppm were assigned to the benzene ring protons of GA. These aromatic signals were absent in pure PAM and became broader and slightly shifted compared with free GA, suggesting that the chemical environment of GA changed after incorporation into the PAM structure. Consistent with the NMR interpretation described in Section 2.3, the signal near 4.7 ppm was not used for structural assignment. Therefore, the ^1^H NMR results, together with FTIR and UV–visible spectra, supported the successful preparation of PAM-GA. D. DPPH radical scavenging activity of PAM and PAM-GA at varying concentrations (0.16–5.00 mg/mL).

E. ABTS radical scavenging activity of PAM and PAM-GA at varying concentrations (0.04–5.00 mg/mL). F. Total antioxidant capacity of PAM and PAM-GA, expressed as mM Trolox equivalent. G. Strain sweep rheology of the PAM-GA hydrogel, showing storage modulus (G′) and loss modulus (G″) as functions of strain. H. Step-strain recovery test of the PAM-GA hydrogel, demonstrating its shear-thinning and self-healing behavior under alternating low (0.1%) and high (100%) strain. I. Apparent viscosity of the PAM-GA hydrogel as a function of shear rate (γ), illustrating shear-thinning properties.

GA has a characteristic absorption peak of the catechol group at 260 nm, and PAM modified by GA has a characteristic absorption peak at 280 nm, while PAM has no such absorption, suggesting that PAM introduces phenolic hydroxyl groups (Figure 5A). In the infrared spectrum, GA, PAM-GA and PAM have their own characteristic absorption peaks at different wave numbers, suggesting that PAM introduces a benzene ring (Figure 5B). The nuclear magnetic resonance hydrogen spectrum showed that GA, PAM-GA and PAM showed different characteristic peaks at the corresponding chemical shifts (Figure 5C). The results of UV, IR and ~^1^H-NMR confirmed the successful synthesis of PAM-GA. The antioxidant properties of PAM-GA were determined by DPPH scavenging rate, ABTS scavenging rate and total antioxidant capacity. The results showed that the antioxidant capacity of PAM-GA was significantly better than that of PAM at the same concentration, and its free radical scavenging and total antioxidant capacity increased with the increase in concentration (Figure 5D–F). Within a certain strain range, the hydrogel storage modulus G′ is stable and can withstand large deformation to maintain a three-dimensional structure. G′ intersects the loss modulus curve at 2000% strain, which is the solid–liquid critical point. Beyond this strain, the blank hydrogel network collapses. In the strain cycle test, after 1–2000% strain change, G′ and G recovered rapidly, confirming its self-healing property. The viscosity curve shows that the hydrogel has shear-thinning characteristics, indicating that it is injectable (Figure 5G–I).

As shown in Figure 5D–F, the antioxidant properties of PAM-GA were determined by DPPH scavenging rate, ABTS scavenging rate and total antioxidant capacity. The results showed that the antioxidant capacity of PAM-GA was significantly better than that of PAM at the same concentration, and its free radical scavenging and total antioxidant capacity increased with the increase in concentration.

### 2.6. Morphological Characterization of Hydrogels

A, B. Scanning electron microscopy (SEM) images of the hydrogel at different magnifications, showing the porous three-dimensional network structure. C. Injectability of the hydrogel demonstrated by extruding it through a syringe needle to form the word “LOVE”, indicating its potential for minimally invasive administration. D. Ex vivo adhesion test of the hydrogel on gastric tissue, showing its ability to adhere to biological tissue. E. Tensile and flexibility tests of the hydrogel, demonstrating its stretchability and conformability on a finger model. F. Adhesion test of the hydrogel on different substrates (tissue, glass, and plastic), illustrating its robust adhesive performance on both biological and non-biological surfaces.

**Figure 6:**
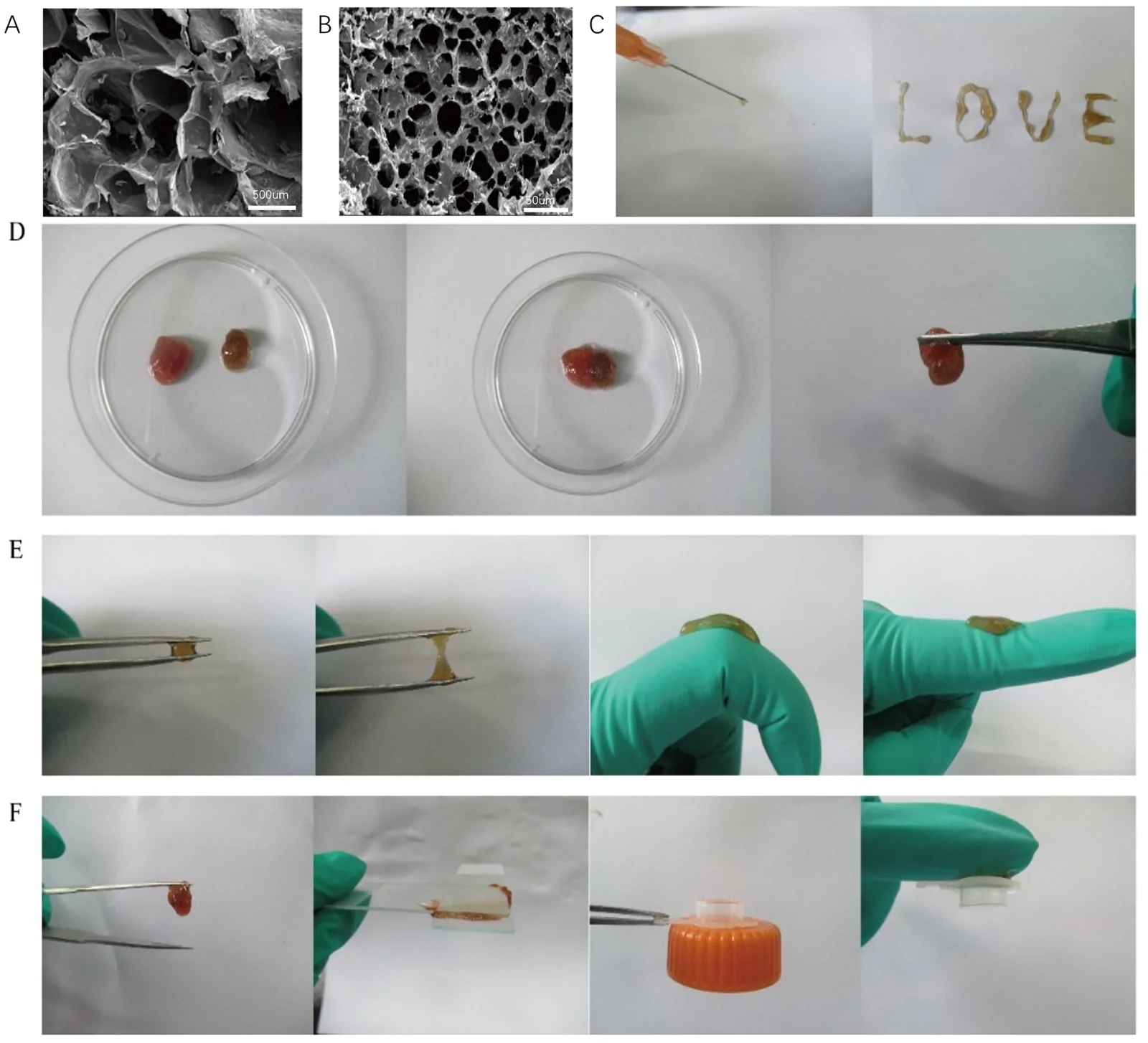
Morphological and functional characterization of the PAM-GA hydrogel. (**A**,**B**) SEM images of the hydrogel showing the porous three-dimensional network structure at different magnifications, with scale bars indicated. (**C**) Injectability of the hydrogel demonstrated by extrusion through a syringe needle to form the word “LOVE”. (**D**) Ex vivo adhesion test showing hydrogel adhesion to biological tissue. (**E**) Tensile and flexibility tests demonstrating the mechanical adaptability of the hydrogel. (**F**) Adhesion test of the hydrogel on different substrates, including tissue, glass, and plastic.

As shown in Figure 6, SEM observation showed that the hydrogel had a porous three-dimensional network structure, and the pore structure became more uniform after protein-loaded nanocapsule incorporation. The hydrogel could be smoothly extruded through a syringe needle to form the word “LOVE”, without obvious blockage or structural collapse, indicating good injectability. After being placed at room temperature for 20 min, the separated hydrogel pieces could reconnect and maintain their integrity under gentle handling, suggesting qualitative self-healing behavior. This observation was consistent with the step-strain rheological recovery results shown in Figure 5H. In addition, the hydrogel could bend and stretch with finger movement and showed adhesion to different substrates, including biological tissue, glass, plastic, and metal, indicating good flexibility and adhesive properties.

### 2.7. In Vitro Protein Release Profile of Hydrogels

**Figure 7:**
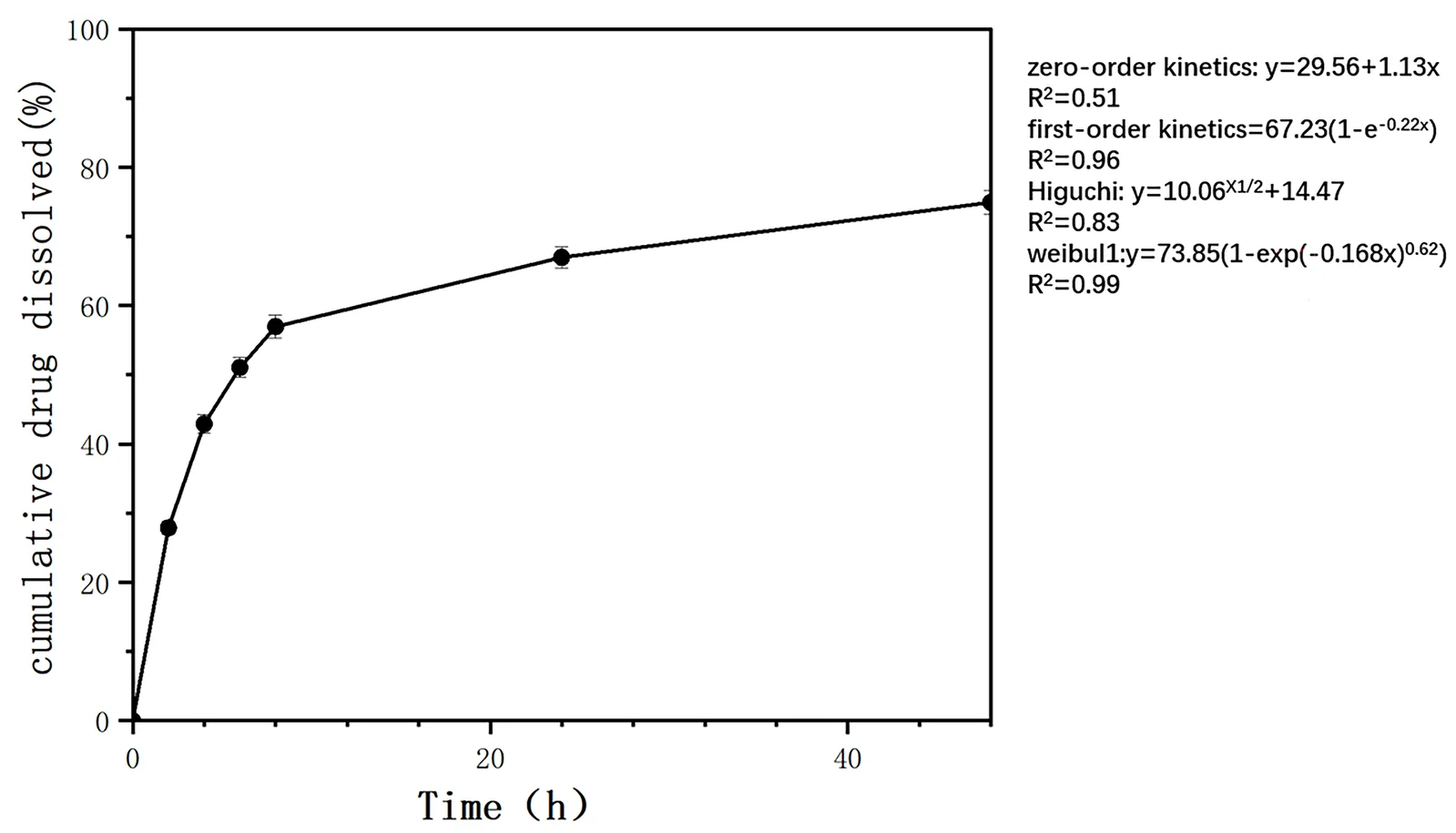
In vitro protein release profile of the hydrogel-based delivery system. The cumulative protein release from the hydrogel was monitored over 48 h. Kinetic fitting was performed using zero-order, first-order, Higuchi, and Weibull models. Data are presented as mean ± SD (*n* = 3).

The in vitro protein release behavior of the hydrogel formulation was evaluated over a 48 h period, and the cumulative release profile is presented in Figure 7. The release experiments were independently repeated three times, and the data are presented as mean ± SD (*n* = 3). The hydrogel exhibited a sustained protein release behavior throughout the 48 h observation period.

The release profile showed a biphasic pattern. During the initial phase (0–6 h), approximately 40–50% of the loaded protein was released, which may be attributed to the diffusion of protein molecules located near the hydrogel surface or loosely associated regions. Subsequently, the release rate gradually decreased, resulting in a slower sustained release phase from 6 to 48 h, with a cumulative release of approximately 70–75%.

To further characterize the release behavior, four kinetic models, including zero-order, first-order, Higuchi, and Weibull models, were applied to fit the release profile. Among these models, the Weibull model showed the highest coefficient of determination (R^2^ = 0.99), followed by the first-order model (R^2^ = 0.96), indicating that the Weibull model provided the best mathematical description of the observed release profile. The Weibull parameter (β = 0.62) suggested a non-ideal release behavior that may involve contributions from diffusion and interactions between the protein and hydrogel matrix.

However, kinetic model fitting should be interpreted cautiously, as the mathematical models describe the overall release behavior rather than directly proving a specific release mechanism. Therefore, the Weibull fitting results were considered supportive evidence for the sustained and complex release characteristics of the hydrogel system, while further studies are required to fully elucidate the detailed release mechanism.

### 2.8. Promoting Chronic Wound Healing Effect

**Figure 8:**
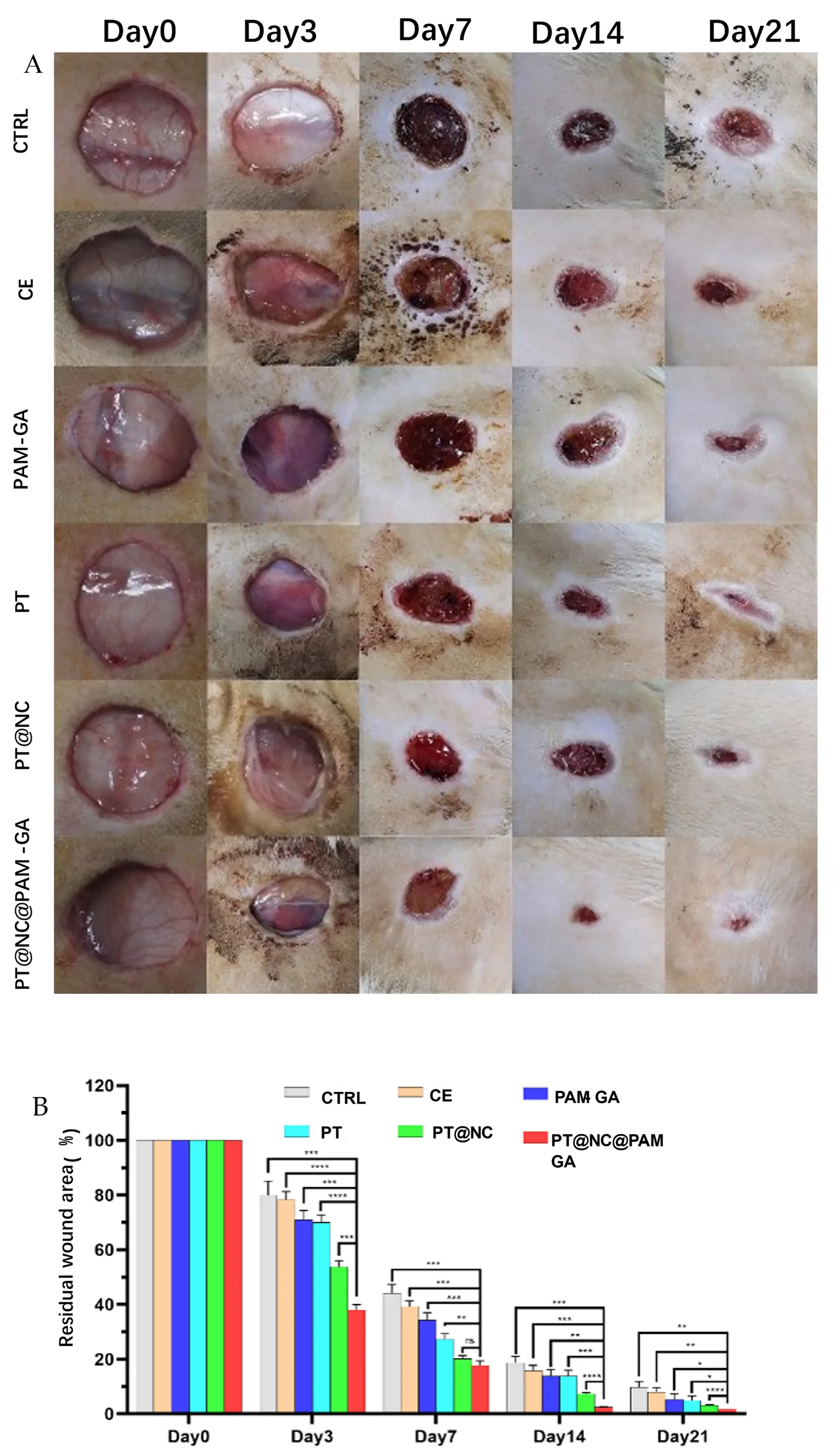
Diabetic rat skin wound healing analysis. (**A**) Representative wound images at different time points. (**B**) Quantitative analysis of residual wound area (%) at different time points. Data are presented as mean ± SD (*n* = 6). Statistical significance: * *p* < 0.05, ** *p* < 0.01, *** *p* < 0.001 and **** *p* < 0.0001.

As shown in Figure 8, all rats developed full-thickness skin defects immediately after surgery (Day 0). During the observation period, different wound-healing patterns were observed among the treatment groups. On Day 3, the CTRL and CE groups exhibited slower wound contraction, with obvious wound areas remaining, whereas the PAM-GA and PT groups showed relatively accelerated wound closure. The PT@NC and PT@NC@PAM-GA groups demonstrated more pronounced wound contraction, with reduced wound areas and improved wound appearance. From Day 14 to Day 21, the wounds in the CTRL and CE groups remained incompletely healed, while the PT@NC and PT@NC@PAM-GA groups exhibited more advanced tissue repair.

The quantitative analysis of wound area demonstrated that the PT@NC@PAM-GA group exhibited the most significant wound repair effect. Figure 8B presents the residual wound area (%) of each group at different time points, which showed a continuous decrease during the healing process. In addition, the wound closure rate was calculated based on the reduction in wound area. The wound closure rate of the PT@NC@PAM-GA group reached 33.56 ± 3.95% on Day 3 and 73.91 ± 3.74% on Day 7, indicating accelerated wound contraction and repair. The PT@NC and PT groups also showed improved wound repair outcomes, characterized by reduced residual wound areas compared with the CTRL and CE groups.

From Day 14 to Day 21, the PT@NC@PAM-GA group showed nearly complete wound closure, accompanied by improved tissue regeneration. Statistical analysis revealed significant differences between the PT@NC@PAM-GA group and the CTRL and CE groups at multiple time points (*p* < 0.001). These results indicate that the nanocapsule hybrid hydrogel effectively accelerated diabetic wound repair.

**Figure 9:**
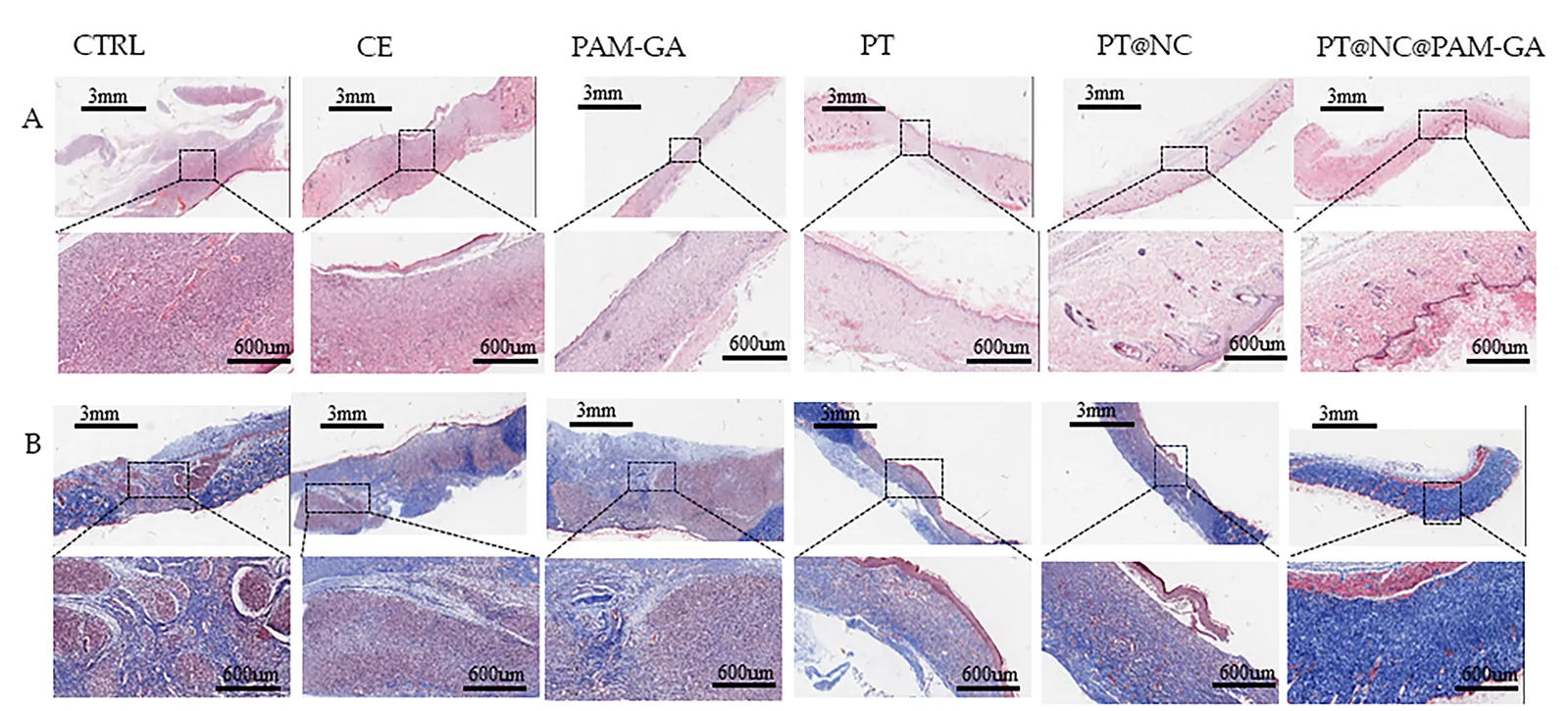
The mechanism of wound healing. (**A**). H&E staining analysis of wounds in different groups. (**B**). MASSON staining analysis of wounds in different groups.

The H & E staining and Masson staining images of the skin wounds of diabetic rats were observed under a microscope, revealing the differences in wound healing and repair between different groups. Figure 9 shows the results on day 21. H & E staining showed that the CTRL group had an obvious inflammatory reaction, more inflammatory cells, incomplete epidermal repair, no obvious angiogenesis, and healing was slow. Compared with the control group, the CE group improved; the wound contracted but still had inflammatory cell infiltration, collagen deposition and angiogenesis were limited; and the wound was not completely healed. In the PAM-GA group, the healing was faster, the tissue repair was good, the inflammatory cells were reduced, the angiogenesis was significantly increased, and the regeneration ability was strong. The healing effect of the PT group and PT@NC group was outstanding; the wound was almost completely healed, the epidermis was reconstructed, the inflammation was reduced, a large amount of new blood vessels were formed, and the collagen fibers were evenly distributed. The healing effect of the PT@NC@PAM-GA group was the best; the epidermal repair was good, the formation of skin appendages (hair follicles, sebaceous glands) was accelerated, and the inflammation was significantly reduced. The collagen fibers in the PT@NC@PAM-GA group were arranged more orderly, and the angiogenesis properties were obvious. Masson staining showed that the collagen deposition in the CTRL group was less, the fiber arrangement was sparse, the wound gap was obvious, and the repair effect was poor. The collagen deposition in the CE group increased and arranged in an orderly manner, and the healing progressed, but the tissue repair was incomplete, and there were still gaps. The collagen deposition in the PAM-GA group increased significantly, arranged in an orderly manner, and the wound healed well with only a small amount of space. The wounds in the PT group were almost filled with collagen, the fibers were dense and neat, the voids were reduced, and the repair effect was better than that of the control group. The collagen deposition in the PT@NC group was the best, the fibers were tight and orderly, and the wound was almost completely healed. The repair effect of the PT@NC@PAM-GA group was the best; the wound was almost healed, the collagen fiber structure was complete, and the tissue regeneration was obvious.

### 2.9. Immunohistochemical Staining Analysis

**Figure 10:**
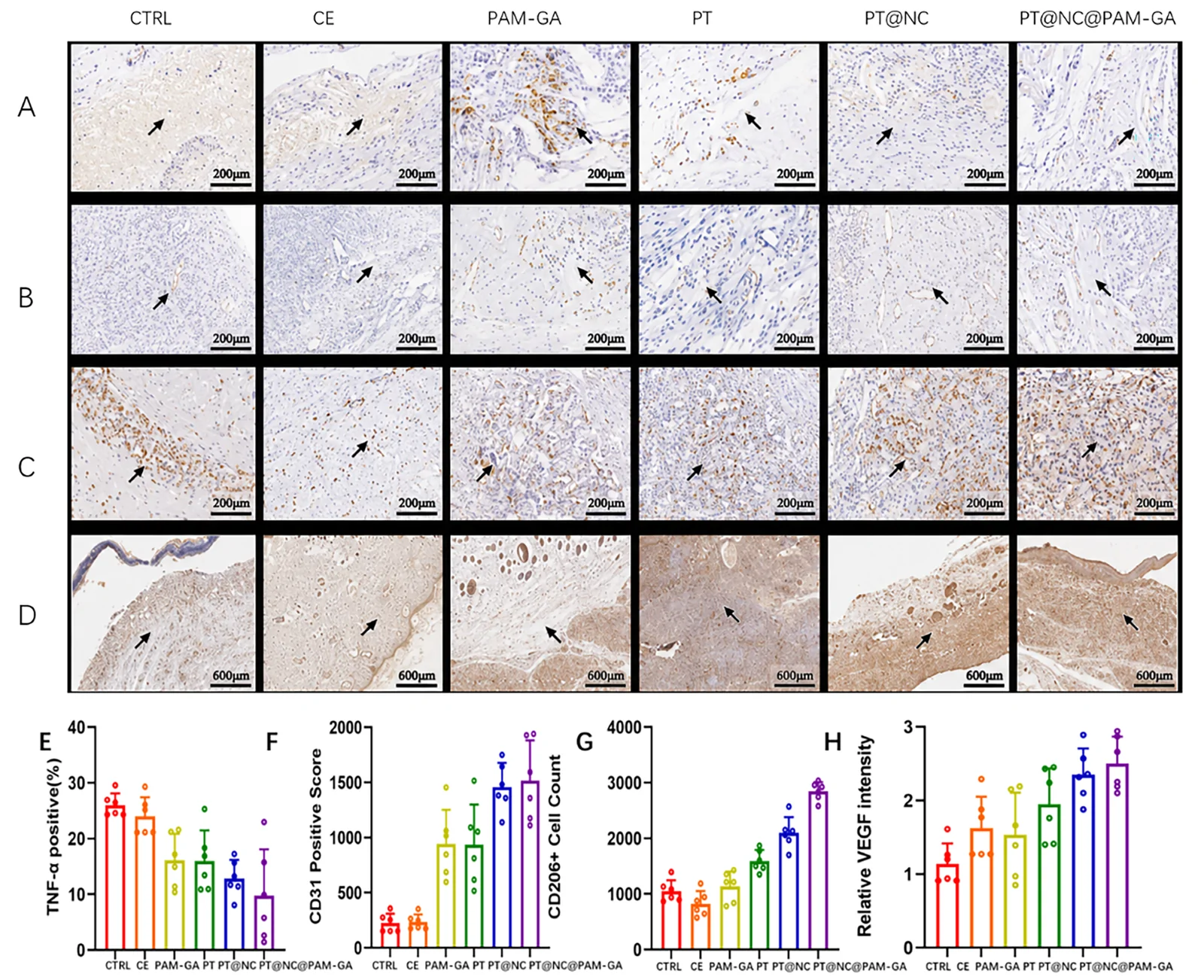
Representative immunohistochemical micrographs showing the expression of key biomarkers in tissue sections. (**A**) TNF-α (pro-inflammatory cytokine), (**B**) CD31 (endothelial cell marker for neovascularization), (**C**) CD206 (M2 macrophage marker), and (**D**) VEGF (angiogenic factor). Black arrows indicate representative positively stained areas/cells for the corresponding biomarkers. Scale bars: 200 μm (**A**–**C**) and 600 μm (**D**). (**E**–**H**) Quantitative analysis of (**E**) TNF-α-positive area percentage, (**F**) CD31-positive microvessel density score, (**G**) CD206-positive cell count, and (**H**) relative VEGF expression intensity. All data are expressed as mean ± standard deviation (SD, *n* = 6).

Figure 10 shows the results of immunohistochemical staining on the 21st day. The results showed that there were significant differences in inflammation, angiogenesis and macrophage infiltration in each treatment group. The expression of TNF-α: the CTRL group had the strongest inflammatory response; the CE and PAM-GA groups decreased, the PT, PT@NC and PT@NC@PAM-GA groups decreased significantly, and the hydrogel group had obvious anti-inflammatory advantages. CD31 is a kind of vascular endothelial marker. The angiogenesis of CTRL and CE groups was insufficient, while that of the PAM-GA, PT, PT@NC and PT@NC@PAM-GA groups was significantly increased, especially in the PT and PT@NC@PAM-GA groups. CD206 is a kind of M2-type macrophage marker. The infiltration of macrophages in the CTRL and CE groups was significant, and the expression of PAM-GA, PT, PT@NC and PT@NC@PAM-GA groups was increased, suggesting that the inflammatory response was inhibited. VEGF is a kind of vascular endothelial growth factor. The expression in the CTRL group was low, and the PT and PT@NC@PAM-GA groups were significantly increased, indicating that the hydrogel promoted angiogenesis.

## 3. Discussion

In this study, we successfully constructed a multifunctional nanocomposite hydrogel system by integrating bioactive proteins extracted from processed medicinal centipede material recorded as Scolopendra subspinipes mutilans with gallic acid (GA), a natural polyphenolic compound commonly found in traditional Chinese medicine. This design was aimed at addressing the persistent clinical challenge of diabetic foot ulcers—a refractory form of chronic wound characterized by delayed healing, persistent inflammation, and impaired angiogenesis [25]. The combination of traditional medicinal components with modern nanotechnology allowed us to develop a hydrogel with enhanced biological efficacy, sustained local delivery properties, and improved physicochemical stability [26]. A comprehensive evaluation through in vitro cell-based assays and in vivo animal experiments demonstrated the hydrogel’s potent therapeutic potential in promoting diabetic wound repair [27]. Specifically, the system effectively overcomes major limitations associated with traditional treatments, such as drug instability and poor bioavailability in the hostile microenvironment of chronic wounds. Moreover, the hydrogel exhibited excellent biocompatibility, multiple therapeutic bioactivities, and controllable protein release behavior, thereby providing a promising new strategy and theoretical basis for the treatment of chronic, non-healing wounds [28,29].

A key feature of this study is the screening and utilization of centipede-derived protein fractions as natural bioactive components for diabetic wound repair. Previous studies have demonstrated that animal-derived bioactive proteins and peptides can participate in tissue repair processes by regulating cell migration, proliferation, inflammation, and extracellular matrix remodeling [30,31]. In addition, tropomyosin-related proteins and calcium-associated regulatory proteins have been reported to participate in cellular regulation processes, suggesting their potential relevance to tissue repair-associated biological functions [32]. In this study, mass spectrometry-based peptide matching identified several protein-related fractions from centipede-derived material, and these fractions were further evaluated through preliminary cellular screening. Among the tested fractions, the tropomyosin-containing fraction and calglandulin-containing fraction exhibited relatively favorable CCK-8-based cellular compatibility/metabolic activity. Considering its overall performance in preliminary screening, the tropomyosin-containing fraction was selected for subsequent nanocapsule construction.

It should be noted that the identified proteins, including actin- and myosin-related proteins, are conserved proteins and are not unique to centipedes. Therefore, the therapeutic effects observed in this study should not be attributed to a single centipede-specific protein, but rather to the combined effects of the screened centipede-derived protein fraction and the nanocapsule-hydrogel delivery system. Further studies using purified proteins, recombinant proteins, and component-specific validation strategies are required to determine the precise contribution of individual proteins to diabetic wound repair.

To further enhance the delivery and stability of these proteins, we encapsulated them into nanocapsules using biocompatible materials. The resulting nanocapsules exhibited uniform particle size, smooth surface morphology, and controlled release kinetics, making them ideal carriers for fragile protein drugs [33]. Importantly, the encapsulation improved protein stability against proteolytic degradation and oxidative damage in the wound environment [34]. In recent years, numerous studies have highlighted the advantages of nanocarrier-based delivery systems in treating chronic wounds, especially diabetic foot ulcers, by significantly prolonging drug half-life, improving tissue penetration, and enhancing therapeutic targeting [35,36]. Our findings support these conclusions and suggest that nanocapsule-assisted delivery is a promising approach to achieving sustained and localized therapeutic effects in complex wound settings [37,38].

The hydrogel matrix itself was engineered using PAM (polyacrylamide) chemically modified with gallic acid, forming a crosslinked three-dimensional network with excellent physicomechanical properties. The incorporation of GA not only retained the desirable traits of PAM, such as injectability and mechanical integrity, but also significantly enhanced the system’s antioxidant capacity and tissue adaptability [39,40]. Gallic acid, as a natural phenolic antioxidant, has been reported to effectively neutralize ROS (reactive oxygen species), reduce oxidative stress, and modulate immune responses in damaged tissues [41]. Additionally, GA can promote angiogenesis by upregulating vascular growth factors and stimulating endothelial cell activity [42]. These properties are especially critical in diabetic wounds, which are often characterized by high oxidative stress, poor vascularization, and chronic inflammation [43].

Animal model experiments further validated the therapeutic efficacy of the developed nanocapsule hybrid hydrogel loaded with protein formulation (PT@NC@PAM-GA). In a streptozotocin-induced diabetic foot ulcer model, the PT@NC@PAM-GA group showed markedly improved wound healing outcomes compared to the control groups. Specifically, PT@NC@PAM-GA application significantly accelerated wound closure, enhanced re-epithelialization, and promoted neovascularization, as evidenced by increased expression of vascular endothelial growth factor (VEGF) and CD31. Moreover, the hydrogel induced greater collagen deposition and more robust granulation tissue formation. It also downregulated key pro-inflammatory cytokines, such as TNF-α, indicating effective suppression of the chronic inflammatory response [44]. These results align well with recent studies on multifunctional hydrogel systems that exert their therapeutic effects through a coordinated anti-inflammatory, pro-angiogenic, and antioxidant mechanism—providing strong support for the clinical translation of such designs in chronic wound care [45,46].

Beyond its biological performance, the composite hydrogel also demonstrated advantageous material characteristics for clinical application. These include self-healing ability, conformability to irregular wound surfaces, and strong adhesion to diverse tissue types. These traits are essential for maintaining intimate contact between the wound bed and therapeutic matrix, ensuring continuous drug delivery and minimizing the risk of secondary injury during dressing changes [47,48]. The hydrogel’s injectable nature further allows for minimally invasive application and complete wound coverage. Such properties are in line with current biomaterials research trends, which emphasize the development of intelligent, adaptable, and highly adhesive hydrogel systems for clinical use [49,50].

Importantly, the different wound-healing effects observed among the treatment groups can be explained by their different structural and functional compositions. The CTRL group lacked both bioactive protein and hydrogel protection, resulting in limited regulation of the diabetic wound microenvironment. The CE and PT groups contained centipede-derived bioactive components, but the absence of a sustained-release carrier may have limited their local retention and therapeutic duration at the wound site. The PAM-GA group provided a protective hydrogel matrix with antioxidant activity and wound coverage, but lacked the protein component that could directly support wound-related cellular activity. The PT@NC group improved protein delivery through nanocapsule encapsulation, but did not provide the same adhesive, antioxidant, and wound-covering microenvironment as the hydrogel matrix. In contrast, PT@NC@PAM-GA integrated the advantages of all components, including sustained protein release from nanocapsules, antioxidant activity from the gallic acid-modified hydrogel, and structural support from the injectable and adhesive hydrogel network. This structural integration explains why PT@NC@PAM-GA showed the most favorable wound-healing performance among the tested groups.

The in vivo results further support this interpretation. Compared with the other groups, PT@NC@PAM-GA accelerated wound closure and promoted more complete tissue regeneration. H&E staining indicated improved re-epithelialization and granulation tissue formation, while Masson staining showed enhanced collagen deposition, suggesting improved extracellular matrix remodeling. The increased expression of CD31 and VEGF indicated enhanced neovascularization, which is essential for nutrient supply and tissue repair in diabetic wounds. Meanwhile, the reduced expression of TNF-α suggested that PT@NC@PAM-GA helped attenuate the excessive inflammatory response. Therefore, the superior wound-healing effect of PT@NC@PAM-GA was not simply due to hydrogel morphology, but resulted from the synergistic combination of bioactive protein delivery, sustained release, antioxidant microenvironment regulation, angiogenesis promotion, collagen remodeling, and inflammation reduction.

Although the present study demonstrated the therapeutic potential of the centipede-derived protein nanocapsule hybrid hydrogel for diabetic wound repair, several limitations should be acknowledged. First, as the bioactive components were derived from processed medicinal centipede material, comprehensive biosafety evaluations, including sterility testing, endotoxin assessment, viral screening, and broader pathogen detection, were not performed in the current study. Therefore, further systematic safety evaluation and quality control of the source material will be necessary before potential clinical translation. Future studies should establish standardized preparation protocols and comprehensive biosafety assessments to facilitate the translational application of this natural-derived therapeutic platform.

## 4. Materials and Methods

### 4.1. Instruments

All chemicals and reagents were of analytical grade and used without further purification unless otherwise stated. Centipede-derived protein was prepared and used as the principal bioactive component. MPC, BIS, NAS, APS, and TEMED were used for the preparation of nanocapsules and hydrogel systems. Superdex 75 Increase 10/300 GL was used for protein purification. Ultrapure water was obtained from a Milli-Q water purification system and used throughout the experiments. Other reagents, buffers, and solvents used in this study were purchased from commercial suppliers.

### 4.2. Drugs and Reagents

The drugs and reagents used in this study are listed in Table 2.

### 4.3. Methods

#### 4.3.1. Extraction, Separation and Characterization of Active Protein

##### Extraction and Purification of Centipede-Derived Protein

The centipede-derived material used in this study was processed medicinal centipede material/extract recorded as Scolopendra subspinipes mutilans, rather than living animals. The source material was used as the starting material for protein extraction and was further processed by aqueous extraction, fractional ethanol precipitation, centrifugation, filtration, chromatographic purification, ultrafiltration or dialysis desalting, and lyophilization. Specific sterility, viral, endotoxin, or broad pathogen screening of the original source material was not performed in the present study; therefore, further systematic biosafety evaluation will be required before translational or clinical application.

Protein identification was performed by mass spectrometry followed by database matching against available UniProt protein entries. Because the reference proteome annotation for Scolopendra subspinipes mutilans is limited, some identified peptides were matched to homologous proteins from closely related centipede species, including *Scolopendra viridis* and *Hemiscolopendra marginata*. Therefore, the species names listed in Table 1 represent the closest available UniProt database matches and do not indicate that these species were used as the source material for extraction.

The source material was used as the starting material for protein extraction and was further processed by aqueous extraction, fractional ethanol precipitation, centrifugation, filtration, chromatographic purification, ultrafiltration or dialysis desalting, and lyophilization. Specific sterility, viral, endotoxin, or broad pathogen screening of the original source material was not performed in the present study; therefore, further systematic biosafety evaluation will be required before translational or clinical application. Centipede-derived protein was extracted using a water extraction and alcohol precipitation method. Briefly, the centipede extract was first prepared by aqueous extraction, followed by ethanol precipitation. After precipitation with 70% ethanol, the supernatant was collected for further fractionation. Subsequently, 95% ethanol was added to the collected supernatant to adjust the final ethanol concentration to 80% (*v*/*v*). The mixture was stirred at 500 rpm for 10 min and then kept at 4 °C overnight. After precipitation, the sample was centrifuged at 8000 rpm for 10 min. The obtained precipitate was redissolved in deionized water or PBS and lyophilized. The remaining supernatant was evaporated to remove ethanol and then lyophilized for further analysis.

The bioactive fraction obtained from 80% ethanol precipitation was further purified using an ÄKTA Pure chromatography system equipped with a Superdex 75 Increase 10/300 GL column. The column was equilibrated and eluted with PBS buffer containing 0.01 M Na_2_HPO_4_/NaH_2_PO_4_ and 0.14 M NaCl. The flow rate was set at 0.5 mL/min, the injection volume was 0.2 mL, and the elution profile was monitored at 280 nm. The purification was performed at room temperature. The main eluted fractions were collected according to the UV absorption peaks, followed by ultrafiltration or dialysis for desalting. The purified fractions were then lyophilized and stored for subsequent experiments.

Protein concentration was determined using the BCA assay according to the manufacturer’s instructions. Briefly, BCA working solution was prepared by mixing reagent A and reagent B at a ratio of 50:1. Then, 0.025 mL of sample solution was mixed with 0.2 mL of BCA working solution and incubated at 37 °C for 30 min. The absorbance was measured at 562 nm using a microplate reader. A standard curve was established using protein standards ranging from 0 to 2000 μg/mL, with R^2^ ≥ 0.98. The protein concentration of each sample was calculated according to the standard curve after correction by the dilution factor.

#### 4.3.2. Preparation and Characterization of Nanocapsules

##### Preparation of Acrylated Centipede-Derived Protein

Centipede-derived protein was acrylated using N-acryloyl succinimide (NAS) before nanocapsule synthesis. Briefly, 20 mg of centipede-derived protein was dissolved in 10 mL of 50 mM HEPES buffer (pH 8.5). NAS was dissolved in anhydrous dimethyl sulfoxide (DMSO) to prepare a 20 mg/mL NAS stock solution. The NAS solution was then added dropwise to the protein solution under stirring, and the reaction was carried out at 4 °C for 2 h.

To optimize the acrylation degree, different NAS feeding amounts were tested. In this study, one-fold NAS was defined as 75 μL of NAS stock solution added to 20 mg of protein in 10 mL of HEPES buffer. Accordingly, two-fold, three-fold, four-fold, five-fold, ten-fold, and twenty-fold NAS feeding amounts corresponded to 150, 225, 300, 375, 750, and 1500 μL of NAS stock solution, respectively. The unmodified protein solution without NAS addition was used as the blank control. After reaction, the acrylated protein solution was purified to remove unreacted NAS and used for subsequent nanocapsule preparation.

The acrylation degree was evaluated using a fluorometric assay. The reacted protein samples were diluted to 0.00781, 0.01563, 0.03125, 0.0625, 0.125, 0.25, 0.5, and 1 mg/mL. The fluorophore was dissolved in anhydrous DMSO to prepare a 3 mg/mL stock solution. Then, 100 μL of each diluted sample was transferred into an opaque 96-well plate, followed by the addition of 30 μL of fluorophore solution. After incubation at 25 °C for 1 h, the fluorescence intensity was measured using a microplate reader at an excitation wavelength of 360 nm and an emission wavelength of 465 nm. The acrylation degree was calculated by comparing the fluorescence intensity of NAS-modified samples with that of the unmodified blank control.

##### Synthesis of Nanocapsules

1 mL of 1 mg·mL^−1^ acrylic tropomyosin, 530 μL of 2-methacryloyloxyethyl phosphorylcholine (MPC) (25%, *w*/*v*) and 104 μL of N,N′-Methylenebisacrylamide (BIS) (10%, *w*/*v*) were added to 50 mM, pH 7.0 phosphate buffer and fully mixed. Free radical polymerization was initiated by adding 32 μL ammonium persulfate (APS, 10%, *w*/*v*) and 10 μL N,N,N′,N′-Tetramethylethylenediamine (TEMED). The reaction was carried out at room temperature (25 °C) for 2 h. After the polymerization reaction, the solution was extensively dialyzed through 1 × phosphate-buffered saline (PBS) to remove unreacted small molecules, and then a 5% sucrose freeze-drying protective agent was added to freeze-dry the dialysis product to obtain solid particles.

##### Morphological Characterization of Nanocapsules

The nanocapsule samples were freeze-dried to obtain powders, which were observed by scanning electron microscopy (SEM) and transmission electron microscopy (TEM). During SEM characterization, the samples were uniformly coated on the conductive silicon wafer. After gold spraying, the surface morphology was recorded by JEOL JSM-6390 LV SEM (JEOL Ltd., Tokyo, Japan) (5 kV accelerated voltage). For TEM analysis, the sample was diluted to 0.1 mg/mL and added to the copper grid. After drying, the internal structure and particle size distribution were observed by JEOL JEM-2100 TEM (JEOL Ltd., Tokyo, Japan) (80 kV accelerated voltage).

##### Structural Characterization of Nanocapsules

UV spectrum: The nanocapsules were dissolved in solvent (5–10 mg/mL) and scanned in the range of 200–350 nm. The participation of reactants was confirmed by characteristic absorption peaks.

Infrared spectroscopy: The nanocapsules were mixed with KBr at a ratio of 1:100, and the functional groups were analyzed using a Fourier transform infrared spectrometer (4000–500 cm, 1, 4 cm, 1 resolution, 64 scans).

Nuclear magnetic resonance spectroscopy: The sample was dissolved in deuterated solvent (D_2_O), and the molecular structure was confirmed by the characteristic signals of 4.7 ppm (phenolic hydroxyl group), 7 ppm (benzene ring), and 3–4 ppm (amino/hydroxyl group) in the ^1^H NMR spectrum.

##### In Vitro Protein Release Profiles of Nanocapsules

The nanocapsules were loaded into a dialysis bag (3kDa) and shaken at 37 °C and 100 rpm with 50 mL PBS (pH 7.4) as the dissolution medium. According to the time interval (1/2/4 h, etc.), 1 mL of dissolution solution was taken, and the same volume of PBS was supplemented. The absorbance was measured by ultraviolet spectrophotometer (280 nm). The release experiment was independently performed three times. The Higuchi model formula, Zero-order model formula, First-order model formula, and Weibull model formula were fitted, and the cumulative release amount was calculated and the curve was drawn.

#### 4.3.3. Preparation and Characterization of Hydrogel

##### Synthesis of Hydrogel

250 mg polyacrylamide (PAM) was dissolved in PBS buffer at pH 5, and 311 mg EDC and 187 mg NHS were added. After 147.5 mg gallic acid was dissolved in ethanol and adjusted to pH 5, PAM solution was added to react for 12 h, and PAM-GA was obtained by dialysis with PBS at pH 5. PAM-GA was dissolved in PBS, and the blank hydrogel was prepared by adding sodium periodate at a mass ratio of 40:1. The drug-loaded hydrogel was prepared by dissolving the nanocapsules in PBS and then adding PAM-GA.

##### Structural Characterization of Hydrogels

The ^1^HNMR spectra of PAM-GA, PAM and GA were measured by nuclear magnetic resonance instrument (600 MHz) (D_2_O as solvent, 4.70 ppm as internal standard). The infrared spectrum from 4000~400 cm^−1^ was measured by an infrared spectrometer. UV-visible spectrum from 800 nm to 200 nm was measured by a UV-visible spectrophotometer.

##### Morphology and Performance Characterization of Hydrogels

Morphology: The hydrogel was rapidly frozen in liquid nitrogen and then freeze-dried for 48 h. The freeze-dried hydrogel was transversely cut into slices approximately 2 mm thick and observed by scanning electron microscopy (SEM). Injectability: The pre-gel solution was loaded into a 1 mL syringe and allowed to form a gel. The hydrogel was then extruded through the syringe to write the word “LOVE” to visually demonstrate its injectability. Deformability: The hydrogel was placed over a finger joint, and its deformability was visually evaluated during repeated bending and extension of the joint. Self-healing: PAM-GA was dissolved in PBS and divided into two portions, which were separately stained with appropriate amounts of rhodamine B and methyl blue. Sodium periodate was subsequently added to each portion as a crosslinking agent to induce hydrogel formation. A half was cut from each of the two differently stained hydrogels, and their freshly cut surfaces were brought into contact with each other in the same Petri dish. After standing, the interface between the two hydrogel halves was visually observed to evaluate their self-healing ability. Adhesion: The adhesive properties of the hydrogel were qualitatively evaluated by allowing it to adhere to plastic, metal, and glass surfaces.The Oxidation Resistance of Hydrogel.

DPPH free radical scavenging rate: DPPH powder dissolved in ethanol solution under dark conditions with a series of concentrations of PAM and PAM-GA solution. The sample groups (sample solution + DPPH solution), blank group (sample solution + ethanol), and control group (DPPH solution + water) were set; a 96-well plate was incubated for 30 min (room temperature, away from light). The absorbance was measured by a microplate reader at 517 nm, according to the formula (1 − (A_sample_ − A_blank_)/A_control_) × 100%, to calculate the clearance rate. A_sample_ represents the absorbance of the sample group, A_blank_ represents the absorbance of the blank group, and A_control_ represents the absorbance of the control group. The free radical scavenging rate was determined by an ABTS kit with a series of concentration solutions. Total antioxidant capacity with a series of concentration solutions was determined using a FRAP kit.

##### Rheological Properties of Hydrogels

The rotational rheometer (MCR92,50 mm parallel plate rotor, clearance 1 mm, 25 °C) was used to measure the critical strain point by: (1) strain amplitude scanning (γ = 0.1~1000%), (2) strain cycle test (strain 1%~2000%), (3) shear-thinning test (shear rate 0.1–1000 s^−1^) to evaluate the rheological properties of hydrogels.

##### In Vitro Protein Release of Hydrogels

The nanocapsules were mixed with the hydrogel precursor solution to form a gel, placed in a dialysis bag, immersed in 20 mL phosphate buffer, and placed in a 37 °C, 100 rpm thermostatic oscillator. The 0.5 mL release medium was taken, and the fresh liquid was replenished. The protein concentration was measured by the BCA method.

#### 4.3.4. In Vitro Cytotoxicity Evaluation

NIH3T3, HaCaT and HUVEC cells were cultured using their complete medium (NIH3T3: 90% high glucose DMEM (Dulbecco’s Modified Eagle Medium), 10% FBS (Fetal Bovine Serum), 1% antibiotics; HaCaT: 90% high glucose RPMI (Roswell Park Memorial Institute medium), 10% FBS, 1% antibiotics. HUVEC: ECM (Endothelial Cell Medium) supplemented with FBS, ECGS (Endothelial Cell Growth Supplement), P/S (Penicillin/Streptomycin) and ECGS/H (Endothelial Cell Growth Supplement plus Heparin) were cultured at 37 °C and 5% CO_2_. Replacing the medium every other day. When the cells grew to 70–80% confluence, they were digested with 0.125% trypsin-EDTA (Ethylenediaminetetraacetic acid) for 3 min, neutralized with serum-containing medium, and resuspended in fresh medium after centrifugation. The cells were adjusted to the appropriate concentration (NIH3T3: 3 × 10^5^ cells/mL, HaCaT: 4 × 10^3^ cells/mL, HUVEC: 3 × 10^4^ cells/mL), and then inoculated into 96-well plates. After 24 h of culture, a protein-containing medium (concentration range from 500 to 16.875 μg/mL) was added and continued incubation for 24 h. After 24 h of treatment, 10% CCK-8 reagent solution was added to each well, followed by incubation at 37 °C for 1 h. The absorbance was then measured at 450 nm using a microplate reader. Cell viability was calculated according to the following equation: Cell viability (%) = [(ODtreated − ODblank)/(ODcontrol − ODblank)] × 100%, where ODtreated represents the absorbance of the treatment group, ODcontrol represents the absorbance of the untreated control group, and ODblank represents the absorbance of medium without cells. It should be noted that the CCK-8 assay mainly reflects cellular metabolic activity based on dehydrogenase activity; therefore, the results were interpreted as CCK-8-based cell viability/metabolic activity rather than direct cell number alone.

#### 4.3.5. Evaluation of Diabetic Wound Healing In Vivo

Thirty-six 5-week-old healthy male SD rats (120 ± 2 g, purchased from Vital River, Beijing, China) were used in this study. All animal experiments were approved by the Ethics Committee of Peking Union Medical College (No. SLXD-20240712021). Before streptozotocin (STZ) injection, rats were fasted for 12 h. STZ was freshly dissolved in 0.1 mol/L citric acid/sodium citrate buffer (pH 4.2–4.5) to prepare a 1% STZ solution. Rats were intraperitoneally injected with STZ at the corresponding dose according to body weight. Four hours after injection, food and glucose-containing water were provided. Blood glucose levels were monitored using a blood glucose meter. Rats with fasting blood glucose ≥ 11.1 mmol/L or random blood glucose ≥ 16.7 mmol/L on two consecutive measurements were considered successfully diabetic. The wound-healing experiment was performed after 2 weeks of blood glucose stabilization.

The diabetic rats were randomly divided into six groups: CTRL, CE, PAM-GA, PT, PT@NC, and PT@NC@PAM-GA. The CTRL group served as the diabetic wound control group. The CE group was treated with crude centipede extract prepared according to the extraction procedure described in Section 4.3.1. The lyophilized extract was dissolved in sterile PBS at the designated concentration and applied to the wound according to the same administration schedule as the other treatment groups. The PAM-GA group was treated with blank gallic acid-modified hydrogel. The PT group was treated with free centipede-derived tropomyosin protein. The PT@NC group was treated with tropomyosin-loaded nanocapsules. The PT@NC@PAM-GA group was treated with a tropomyosin-loaded nanocapsule hybrid PAM-GA hydrogel.

After anesthesia and hair removal, the dorsal skin was disinfected, and a 12 mm full-thickness circular wound was created on the back of each rat. Different treatments were applied to the wound area according to the experimental grouping, and the dressing was changed every 2 days. Wound images were recorded on days 3, 7, 11, 15, and 21. Wound areas were analyzed using ImageJ software (version 1.54f, National Institutes of Health, Bethesda, MD, USA). The residual wound area was calculated as the percentage of the remaining wound area relative to the initial wound area: Residual wound area (%) = (S_t_/S_0_) × 100%, where S_0_ represents the initial wound area and S_t_ represents the wound area at each time point. The wound closure rate was additionally calculated based on the reduction in wound area using the following formula: Wound closure rate (%) = [(S_0_ − S_t_)/S_0_] × 100%, where S_0_ represents the initial wound area and S_t_ represents the wound area at each time point.

On day 21, wound tissue samples were collected from each group for histological and immunohistochemical analyses. The harvested tissues were divided along the wound center. One part was stored at −80 °C for further analysis, and the other part was fixed in 4% paraformaldehyde, dehydrated through a graded ethanol series, embedded in paraffin, and sectioned into 4-μm-thick slices.

For hematoxylin and eosin (H&E) staining, the tissue sections were deparaffinized, rehydrated, stained with hematoxylin for nuclear staining, and then stained with eosin for cytoplasmic staining. After dehydration and clearing, the sections were mounted and observed under a light microscope to evaluate epidermal regeneration, inflammatory infiltration, and granulation tissue formation.

For Masson’s trichrome staining, the sections were deparaffinized, rehydrated, stained with Weigert’s iron hematoxylin, followed by Biebrich scarlet-acid fuchsin staining, differentiated in phosphomolybdic-phosphotungstic acid solution, and finally stained with aniline blue. Collagen fibers were stained blue and analyzed to evaluate collagen deposition and extracellular matrix remodeling.

For immunohistochemical staining, the sections were deparaffinized, rehydrated, and subjected to antigen retrieval. After blocking with serum or bovine serum albumin, the sections were incubated with primary antibodies against wound-healing-related markers, including CD206, CD31, VEGF, and TNF-α, overnight at 4 °C. After washing, the sections were incubated with horseradish peroxidase-conjugated secondary antibodies, followed by DAB chromogen development. The nuclei were counterstained with hematoxylin. Positive staining was observed under a light microscope, and the positive staining area or staining intensity was quantitatively analyzed using ImageJ software.

#### 4.3.6. Statistical Analysis

All quantitative data were expressed as mean ± standard deviation (SD). Statistical analyses were performed using GraphPad Prism 9.0 software (GraphPad Software, Boston, MA, USA). For comparisons among multiple groups, one-way analysis of variance (ANOVA) followed by the least significant difference (LSD) post hoc test was performed. A value of *p* < 0.05 was considered statistically significant. Statistical significance was indicated as * *p* < 0.05, ** *p* < 0.01, and *** *p* < 0.001.

## 5. Conclusions

In summary, the nanocomposite hydrogel based on centipede protein-loaded nanocapsules and gallic acid-modified polymer matrix significantly accelerated the healing of diabetic foot wounds by coordinating the regulation of inflammation, angiogenesis and oxidative stress. As the main bioactive component, centipede protein may promote wound repair by regulating the inflammatory microenvironment, promoting fibroblast activity and enhancing tissue regeneration. The encapsulation in the nanocapsules can not only improve the stability and bioavailability of the protein, but also can be continuously released at the wound site, thereby prolonging its therapeutic effect and improving the local utilization rate. In addition, the nano-delivery system facilitates interaction with wound tissue and enhances the biological activity of the encapsulated protein. At the same time, the gallic acid-modified hydrogel matrix provides antioxidant protection and a favorable moist environment for tissue repair. The synergistic integration of bioactive centipede protein, nanocapsule-mediated delivery and multifunctional hydrogel dressings ultimately contributes to enhanced wound closure and tissue remodeling. Therefore, this study provides a promising strategy for the development of natural bioactive wound dressings and provides new insights into the treatment of diabetic foot ulcers. Future studies should further explore the molecular mechanism of centipede protein-mediated tissue repair and evaluate the clinical transformation potential of the nanocomposite hydrogel system.

## Figures and Tables

**Table 1:** Protein-related components assigned by mass spectrometry peptide matching and UniProt database comparison.

Accession	Name	Description	MW [kDa]
A0A4D5R7V3	Calglandulin	Putative Calglandulin OS = *Scolopendra viridis* OX = 118,503 PE = 3 SV = 1	27.5
A5D6I1	Tropomyosin	Tropomyosin OS = *Scolopendra* sp. YSL-2003 OX = 258,331 PE = 2 SV = 1	32.8
A0A4D5R9D3	Myosin light chain 2	Myosin light chain 2 OS = *Scolopendra viridis* OX = 118,503 PE = 3 SV = 1	19.8
A0A4D5R8T8	Actin	Actin OS = *Scolopendra viridis* OX = 118,503 PE = 3 SV = 1	41.7
A0A646QGK4	Nucleoredoxin-like protein 2	Nucleoredoxin-like protein 2 OS = *Hemiscolopendra marginata* OX = 943,146 PE = 3 SV = 1	20.3
A0A4D5R9Z1	Glyceraldehyde-3-phosphate dehydrogenase	Glyceraldehyde-3-phosphate dehydrogenase OS = *Scolopendra viridis* OX = 118,503 PE = 3 SV = 1	36.1

**Table 2:** Drugs and reagents used in this study.

75% ethanol	Sigma-Aldrich, St. Louis, MO, USA
95% ethanol	Sigma-Aldrich, St. Louis, MO, USA
0.22 um filter membrane	Sigma-Aldrich, St. Louis, MO, USA
Phosphate-buffered solution	Beijing Solarbio Science & Technology Co., Ltd., Beijing, China
3 kDa ultrafiltration tube	Millipore, Burlington, MA, USA
Deionized water	Milli-Q water purification system, Millipore, Burlington, MA, USA
Trypsin	Sigma-Aldrich, St. Louis, MO, USA
CCK-8 Cell Activity Assay Kit	Beyotime Biotechnology, Shanghai, China
DMEM	Gibco, Grand Island, NY, USA
Fetal bovine serum	Gibco, Grand Island, NY, USA
Penicillin-streptomycin solution	Gibco, Grand Island, NY, USA
BCA kit	Wuhan YaKeyin Biotechnology Co., Ltd., Wuhan, China
Polyacrylamide (PAM)	Sigma-Aldrich, St. Louis, MO, USA
Gallic acid (GA)	Sigma-Aldrich, St. Louis, MO, USA
Ammonium persulfate (APS)	Sigma-Aldrich, St. Louis, MO, USA
Tetramethylethylenediamine (TEMED)	Sigma-Aldrich, St. Louis, MO, USA
N-acryloylsuccinimide (NAS)	Sigma-Aldrich, St. Louis, MO, USA
Polyvinyl alcohol (PVA)	Aladdin, Shanghai, China
Sodium periodate	Sigma-Aldrich, St. Louis, MO, USA
Rodamine B	Shanghai Aladdin Biochemical Technology Co., Ltd., Shanghai, China
DPPH	Beijing Solarbio Science & Technology Co., Ltd., Beijing, China
ABTS free radical scavenging kit	Beijing Solarbio Science & Technology Co., Ltd., Beijing, China
Total Antioxidant Capacity (FRAP) Kit	Beyotime Biotechnology, Shanghai, China
SD rats	Veton Liwa Co., Ltd., Beijing, China
12 mm skin puncher	Nanjing Qiangke Biotechnology Co., Ltd., Nanjing, China
Mouse shaving machine	Nanjing Qiangke Biotechnology Co., Ltd., Nanjing, China
Tissue fixative (4%)	Beyotime Biotechnology, Shanghai, China
Paraffin, hematoxylin-eosin staining solution	Beyotime Biotechnology, Shanghai, China
Streptozotocin (STZ)	Shanghai Yuanye Bio-Technology Co., Ltd., Shanghai, China
Tegaderm wound dressing	3M, St. Paul, MN, USA
Masson Trichrome Staining Kit	Beyotime Biotechnology, Shanghai, China
CD206 immunohistochemical staining reagent	Abcam, Cambridge, UK
CD31 immunohistochemical staining reagent	Abcam, Cambridge, UK
VEGF immunohistochemical staining reagent	Abcam, Cambridge, UK
TNF-α immunohistochemical staining reagent	Abcam, Cambridge, UK

## Data Availability

The original contributions presented in this study are included in the article and supplementary material. Further inquiries can be directed to the corresponding authors. During the preparation and revision of this manuscript, AI-assisted tools were used to help improve English language expression, grammar, and clarity. No AI tools were used to generate original experimental data, perform data analysis, draw scientific conclusions, or replace the authors’ intellectual contributions. The authors carefully reviewed and edited all AI-assisted content and take full responsibility for the final manuscript.

## Supplementary Materials

The following supporting information can be downloaded at: https://www.mdpi.com/article/10.3390/ph19081289/s1, Figure S1: Spectroscopic characterization of the tropomyosin-loaded nanocapsule system. (A) FTIR spectra of the nanocapsule product and related reaction components. (B) UV–visible absorption spectra of the reaction components and tropomyosin-loaded nanocapsule system. (C) ^1^H NMR spectra recorded in D_2_O for structural characterization of the nanocapsule system.

## Abbreviations

DFUs	Diabetic Foot Ulcers
PAM-GA	Polyacrylamide-Gallic Acid
DPPH	2,2-Diphenyl-1-picrylhydrazyl
ABTS	2,2′-Azino-bis(3-ethylbenzothiazoline-6-sulfonic acid)
SEM	Scanning Electron Microscopy
NMR	Nuclear Magnetic Resonance
IR	Infrared
UV	Ultraviolet
CCK-8	Cell Counting Kit-8
ROS	Reactive Oxygen Species
VEGF	Vascular Endothelial Growth Factor
TNF-α	Tumor Necrosis Factor-alpha
CD31	Cluster of Differentiation 31
CD206	Cluster of Differentiation 206
BCA	Bicinchoninic Acid Protein Assay
PBS	Phosphate-Buffered Saline
P/S	Penicillin/Streptomycin
ECGS	Endothelial Cell Growth Supplement
DMEM	Dulbecco’s Modified Eagle Medium
FBS	Fetal Bovine Serum
ECGS/H	Endothelial Cell Growth Supplement plus Heparin
AGEs	Advanced glycation end products
PT	Centipede-derived tropomyosin protein
NC	Nanocapsule
CE	Centipede extract
GA	Gallic acid
NAS	N-acryloylsuccinimide
MPC	2-Methacryloyloxyethyl phosphorylcholine
BIS	N,N′-Methylenebisacrylamide
APS	Ammonium persulfate
TEMED	N,N,N′,N′-Tetramethylethylenediamine
STZ	Streptozotocin

## References

[1] Zhang X., Tao J., Gong S., Yu X., Shao S. (2024). Effects of Recombinant Human Granulocyte/Macrophage Colony-Stimulating Factor on Diabetic Lower Extremity Ulcers: Case Series of Nine Patients. Diabetes Metab. Syndr. Obes. Targets Ther..

[2] McDermott K., Fang M., Boulton A.J., Selvin E., Hicks C.W. (2023). Etiology, Epidemiology, and Disparities in the Burden of Diabetic Foot Ulcers. Diabetes Care.

[3] Armstrong D.G., Tan T.-W., Boulton A.J.M., Bus S.A. (2023). Diabetic Foot Ulcers: A Review. JAMA.

[4] Xiang Z., Cai R.P., Xiao Y., Huang Y.C. (2024). Single-cell sequencing technology in diabetic wound healing: New insights into the progenitors-based repair strategies. World J. Stem Cells.

[5] Dutta A., Bhansali A., Rastogi A. (2021). Early and Intensive Glycemic Control for Diabetic Foot Ulcer Healing: A Prospective Observational Nested Cohort Study. Int. J. Low. Extrem. Wounds.

[6] Lazzarini P.A., Armstrong D.G., Crews R.T., Gooday C., Jarl G., Kirketerp-Moller K., Viswanathan V., Bus S.A. (2023). Effectiveness of offloading interventions for people with diabetes-related foot ulcers: A systematic review and meta-analysis. Diabetes Metab. Res. Rev..

[7] Fareed I.A., Alibrahimi S., Nihad I. (2025). Study Effect of Carbon Dioxide Water Bath Therapy (Carbothera) in Treatment of a Patient With Diabetic Foot Which They Are Not Response to Convention Treatment Such as Daily Dressing, Debridement, Antibiotic, and Laser Therapy in Our Diabetic and Endocrine Center. Endocr. Pract..

[8] Senneville E., Gachet B., Blondiaux N., Robineau O. (2023). Do Anti-Biofilm Antibiotics Have a Place in the Treatment of Diabetic Foot Osteomyelitis?. Antibiotics.

[9] Estelle E., Nestoras M. (2018). Update on management of diabetic foot ulcers. Ann. N. Y. Acad. Sci..

[10] Feitosa Y.S., Cabral B.V.B., Lima G.D.S., Gomes E.B., Sousa G.J.B., Moreira T.M.M., Pereira M.L.D. (2025). Surgical treatment of diabetic foot: A spatial analysis, Ceará, Brazil, 2016–2021. Epidemiol. Serv. Saude.

[11] Bus S.A., Armstrong D.G., Crews R.T., Gooday C., Jarl G., Kirketerp-Moller K., Viswanathan V., Lazzarini P.A. (2023). Guidelines on offloading foot ulcers in persons with diabetes (IWGDF 2023 update). Diabetes Metab. Res. Rev..

[12] Wendland D.M., Altenburger E.A., Swen S.B., Haan J.D. (2024). Diabetic Foot Ulcer beyond Wound Closure: Clinical Practice Guideline. Phys. Ther..

[13] Yorke E., Boima V., Ganu V., Tetteh J., Twumasi L., Ekem-Ferguson G., Kretchy I., Mate-Kole C.C. (2023). The mediating role of quality of life on depression and medication adherence among patients with type 2 diabetes mellitus: A cross-sectional study. Health Sci. Rep..

[14] McCarthy M., Yates T., Webb D., Game F., Gray L., Davies M.J. (2020). Health impacts of seated arm ergometry training in patients with a diabetic foot ulcer: Protocol for a randomised controlled trial. BMJ Open.

[15] Hawkins B.K., Barnard M., Barber K.E., Stover K.R., Cretella D.A., Wingler M.J.B., Wagner J.L. (2021). Diabetic Foot Infections: A Microbiologic Review. Foot.

[16] Kale D., Karande G., Datkhile K. (2023). Diabetic Foot Ulcer in India: Aetiological Trends and Bacterial Diversity. Indian J. Endocrinol. Metab..

[17] Evstratova E., Yatsenko E., Baranovskii D., Klabukov I. (2024). Effectiveness of Stem Cell Therapy for Diabetic Foot Ulcers: Cell Therapy Alone is Not Enough for Effective Management of Chronic Wounds. Int. J. Low. Extrem. Wounds.

[18] Mustafa A., Simay A., Hamdullah Y., Yildirim E., Ali M.B., Hikmet E.G., Yasin G., Yilmaz K.B. (2025). Autologous adipose-derived tissue stromal vascular fraction and intralesional epidermal growth factor combined application in patients with diabetic foot. J. Wound Care.

[19] Magruder M.L., Yao V.J., Rodriguez A.N., Ng M.K., Piuzzi N.S., Mont M.A. (2023). History of Diabetic Foot Ulcer is Associated with Increased Risk of Prosthetic Joint Infection and Sepsis After Total Joint Arthroplasty. J. Arthroplasty.

[20] Liu E., Gao H., Zhao Y., Pang Y., Yao Y., Yang Z., Zhang X., Wang Y., Yang S., Ma X. (2022). The potential application of natural products in cutaneous wound healing: A review of preclinical evidence. Front. Pharmacol..

[21] Han Y., Kamau P.M., Lai R., Luo L. (2022). Bioactive Peptides and Proteins from Centipede Venoms. Molecules.

[22] Lee Y.S., Lee Y.J., Ha I.H. (2025). Therapeutic Potential of Scolopendra subspinipes: A Comprehensive Scoping Review of Its Bioactive Compounds, Preclinical Pharmacology, and Clinical Applications. Toxins.

[23] Du L., Lu H., Xiao Y., Guo Z., Li Y. (2024). Protective effect and pharmacokinetics of dihydromyricetin nanoparticles on oxidative damage of myocardium. PLoS ONE.

[24] Azam F., Ahmad F., Ahmad S., Zafar M.S., Ulker Z. (2022). Preparation and Characterization of Alginate Hydrogel Fibers Reinforced by Cotton for Biomedical Applications. Polymers.

[25] Chen H., Zheng T., Wu C., Wang J., Ye F., Cui M., Sun S., Zhang Y., Li Y., Dong Z. (2022). A Shape-Adaptive Gallic Acid Driven Multifunctional Adhesive Hydrogel Loaded with Scolopin2 for Wound Repair. Pharmaceuticals.

[26] Hou Y., Huang H., Gong W., Wang R., He W., Wang X., Hu J. (2022). Co-assembling of natural drug-food homologous molecule into composite hydrogel for accelerating diabetic wound healing. Biomater. Adv..

[27] Xiong G., Chen Q., Wang Q., Wang X., Xiao Y., Jin L., Yan K., Zhang X., Hu F. (2024). Multifaceted role of nanocomposite hydrogels in diabetic wound healing: Enhanced biomedical applications and detailed molecular mechanisms. Biomater. Sci..

[28] Huang C., Dong L., Zhao B., Lu Y., Huang S., Yuan Z., Luo G., Xu Y., Qian W. (2022). Anti-inflammatory hydrogel dressings and skin wound healing. Clin. Transl. Med..

[29] Yang X., He S., Wang J., Liu Y., Ma W., Yu C.-Y., Wei H. (2023). Hyaluronic acid-based injectable nanocomposite hydrogels with photo-thermal antibacterial properties for infected chronic diabetic wound healing. Int. J. Biol. Macromol..

[30] Deptuła M., Sawicka J., Sass P., Sosnowski P., Karpowicz P., Zawrzykraj M., Wardowska A., Tymińska A., Dzierżyńska M., Pietralik-Molińska Z. (2023). Development and evaluation of RADA-PDGF2 self-assembling peptide hydrogel for enhanced skin wound healing. Front. Pharmacol..

[31] Fan X., Ye J., Zhong W., Shen H., Li H., Liu Z., Bai J., Du S. (2024). The Promoting Effect of Animal Bioactive Proteins and Peptide Components on Wound Healing: A Review. Int. J. Mol. Sci..

[32] Wilken M.B., Fonar G., Qiu R., Bennett L., Tober J., Nations C., Pavani G., Tsao V., Garifallou J., Petit C. (2024). Tropomyosin 1 deficiency facilitates cell state transitions and enhances hemogenic endothelial cell specification during hematopoiesis. Stem Cell Rep..

[33] Martins J.R.P., de Aguiar A.L.L., Nogueira K.A.B., Filho A.J.U.B., Moreira T.D.S., Araújo M.L.H., Pessoa C., Eloy J.O., Junior I.J.D.S., Petrilli R. (2023). Nanoencapsulation of R-phycoerytrin extracted from S. filiformis improves protein stability and enables its biological application as a fluorescent dye. J. Microencapsul..

[34] Du W., Du S., Dong X., Bai H., Jiang J., Hao S., Yang F., Xiao Q., Zhang B., Ge J. (2023). Biodegradable silica nanocapsules enable efficient nuclear-targeted delivery of native proteins for cancer therapy. Biomaterials.

[35] Zhang W., Wang L., Guo H., Chen L., Huang X. (2022). Dapagliflozin-Loaded Exosome Mimetics Facilitate Diabetic Wound Healing by HIF-1α-Mediated Enhancement of Angiogenesis. Adv. Healthc. Mater..

[36] Liu M., Wei X., Zheng Z., Li Y., Li M., Lin J., Yang L. (2023). Recent Advances in Nano-Drug Delivery Systems for the Treatment of Diabetic Wound Healing. Int. J. Nanomed..

[37] Jiang T., Li Q., Qiu J., Chen J., Du S., Xu X., Wu Z., Yang X., Chen Z., Chen T. (2022). Nanobiotechnology: Applications in Chronic Wound Healing. Int. J. Nanomed..

[38] Cao Z., Liu J., Yang X. (2024). Deformable nanocarriers for enhanced drug delivery and cancer therapy. Exploration.

[39] Jongprasitkul H., Parihar V.S., Turunen S., Kellomäki M. (2023). pH-Responsive Gallol-Functionalized Hyaluronic Acid-Based Tissue Adhesive Hydrogels for Injection and Three-Dimensional Bioprinting. ACS Appl. Mater. Interfaces.

[40] Sanshita S., Pahal S., Ghate V., Singh I. (2023). Novel bio-inspired microneedles for wound healing application. Expert Opin. Drug Deliv..

[41] Kumar S., Saharan R., Saini V.D., Saini A. (2025). Gallic Acid: A Wonderful Remedy in Medicinal Field. Curr. Tradit. Med..

[42] Das A., Nikhil A., Shiekh P.A., Yadav B., Jagavelu K., Kumar A. (2024). Ameliorating impaired cardiac function in myocardial infarction using exosome-loaded gallic-acid-containing polyurethane scaffolds. Bioact. Mater..

[43] Diao W., Li P., Jiang X., Zhou J., Yang S. (2023). Progress in copper-based materials for wound healing. Wound Repair Regen..

[44] Ni Z., Yu H., Wang L., Huang Y., Lu H., Zhou H., Liu Q. (2022). Multistage ROS-Responsive and Natural Polyphenol-Driven Prodrug Hydrogels for Diabetic Wound Healing. ACS Appl. Mater. Interfaces.

[45] Liu H., He L. (2025). Intelligent hydrogel-based dressings for treatment of chronic diabetic wounds. World J. Diabetes.

[46] Pontremoli C., Pagani M., Maddalena L., Carosio F., Vitale-Brovarone C., Fiorilli S. (2021). Polyelectrolyte-Coated Mesoporous Bioactive Glasses via Layer-by-Layer Deposition for Sustained Co-Delivery of Therapeutic Ions and Drugs. Pharmaceutics.

[47] Wang P., Wu J., Xiao X., Fan Y., Han X., Sun Y. (2024). Engineering Injectable Coassembled Hydrogel by Photothermal Driven Chitosan-Stabilized MoS2 Nanosheets for Infected Wound Healing. ACS Nano.

[48] Wang L., Zhao Z., Dong J., Li D., Dong W., Li H., Zhou Y., Liu Q., Deng B. (2023). Mussel-Inspired Multifunctional Hydrogels with Adhesive, Self-Healing, Antioxidative, and Antibacterial Activity for Wound Healing. ACS Appl. Mater. Interfaces.

[49] Bian S., Hao L., Qiu X., Wu J., Chang H., Kuang G., Zhang S., Hu X., Dai Y., Zhou Z. (2022). An Injectable Rapid-Adhesion and Anti-Swelling Adhesive Hydrogel for Hemostasis and Wound Sealing. Adv. Funct. Mater..

[50] Zhang M., Yang Y., Li M., Shang Q., Xie R., Yu J., Shen K., Zhang Y., Cheng Y. (2023). Toughening Double Network Hydrogels by Polyelectrolytes. Adv. Mater..

