# Supplementary material for: Centipede Protein-Laden Natural Nanocapsule Hybrid Hydrogel Mediates Sustained Bioactive Release for Synergistic Regeneration of Diabetic Foot Ulcers
Source: Pharmaceuticals (Basel). 2026 Aug 14;19(8):1289. doi: 10.3390/ph19081289 (PMC13516529; doi:10.3390/ph19081289)
Supplement: Supplementary file 1 [file pharmaceuticals-19-01289-s001.zip › pharmaceuticals-4421686-supplementary.pdf]

Supporting Information

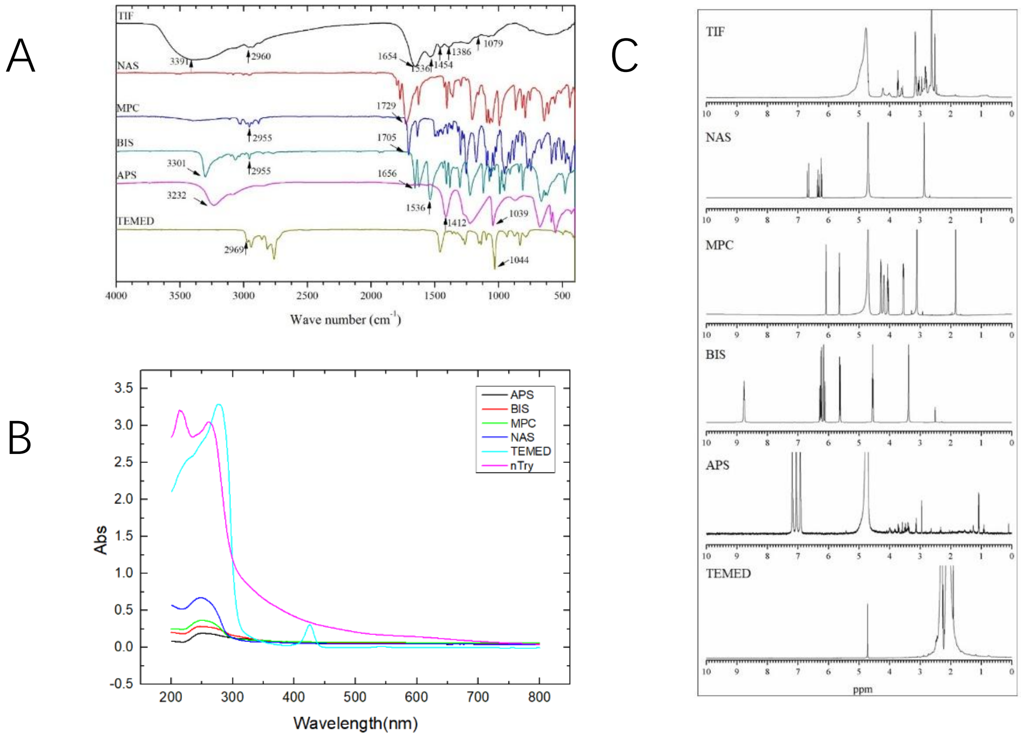

Figure S1. Spectroscopic characterization of the tropomyosin-loaded nanocapsule system. (A) FTIR spectra of the nanocapsule product and related reaction components. (B) UV–visible absorption spectra of the reaction components and tropomyosin-loaded nanocapsule system. (C) ^{1}H NMR spectra recorded in D_2O for structural characterization of the nanocapsule system.
